# Supplementary material for: T-type Calcium Channels Determine the Vulnerability of Dopaminergic Neurons to Mitochondrial Stress in Familial Parkinson Disease
Source: Stem Cell Reports. 2018 Oct 18;11(5):1171–84. doi: 10.1016/j.stemcr.2018.09.006 (PMC6234903; doi:10.1016/j.stemcr.2018.09.006)
Supplement: Document S1. Supplemental Experimental Procedures, Figures S1–S6, and Tables S1 and S2 [file mmc1.pdf]

Stem Cell Reports, Volume *11*

Supplemental Information

T-type Calcium Channels Determine the Vulnerability of Dopaminergic Neurons to Mitochondrial Stress in Familial Parkinson Disease

Yoshikuni Tabata, Yoichi Imaizumi, Michiko Sugawara, Tomoko Andoh-Noda, Satoe Banno, MuhChyi Chai, Takefumi Sone, Kazuto Yamazaki, Masashi Ito, Kappei Tsukahara, Hideyuki Saya, Nobutaka Hattori, Jun Kohyama, and Hideyuki Okano

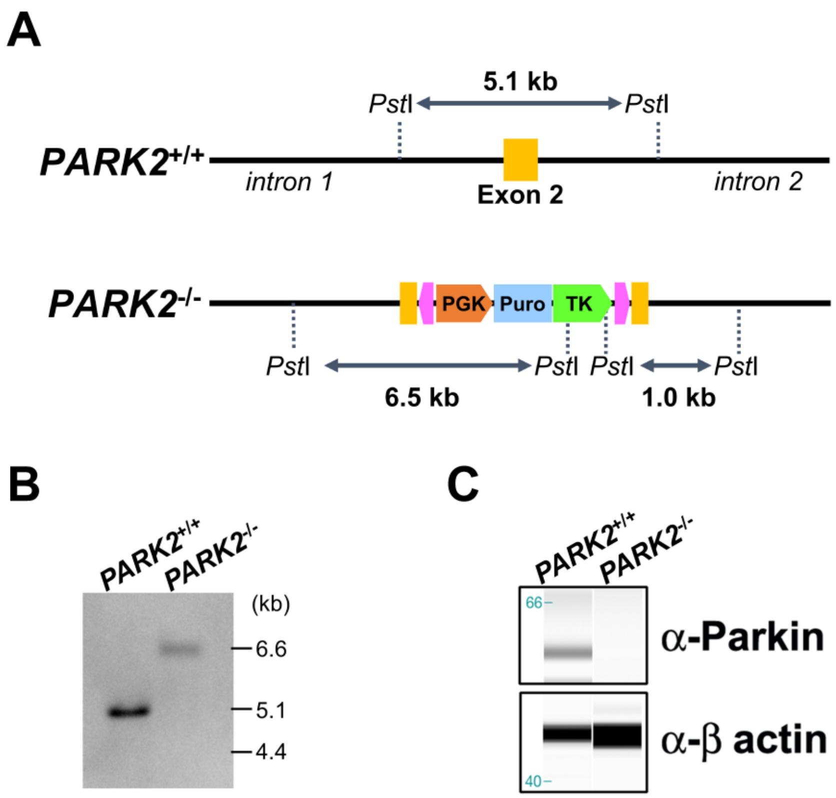
A
5.1 kb
PstI
PstI
PARK2+/+
intron 1
Exon 2
intron 2
PARK2-/-
PGK
Puro
TK
PstI
PstI
PstI
PstI
6.5 kb
1.0 kb
B
PARK2+/+
PARK2-/-
(kb)
6.6
5.1
4.4
C
PARK2+/+
PARK2-/-
66
40
α-Parkin
α-β actin

Figure S1. Generation of Isogenic *PARK2*$^{-/-}$ iPSC-derived Neural Progenitor Cells (Related to Figure 1)

(A) Design of *PARK2* gene knock-out by CRISPR-Cas9 method and construction of plasmid vectors. To generate *PARK2*-deficient iPSC line, we used CRISPR-Cas9 and a targeting donor vector with puromycin resistant gene. After selection with puromycin, isogenic *PARK2*$^{-/-}$ iPSC lines (B7PA21) was identified. (B) To confirm homologous recombination in *PARK2* gene locus, Southern-blotting analysis was used. *PARK2*$^{-/-}$ showed correct 6.5 kb band. (C) The expression of target proteins was determined by the capillary electrophoresis immunoassay using the Wes size-based Simple Western system (ProteinSimple).

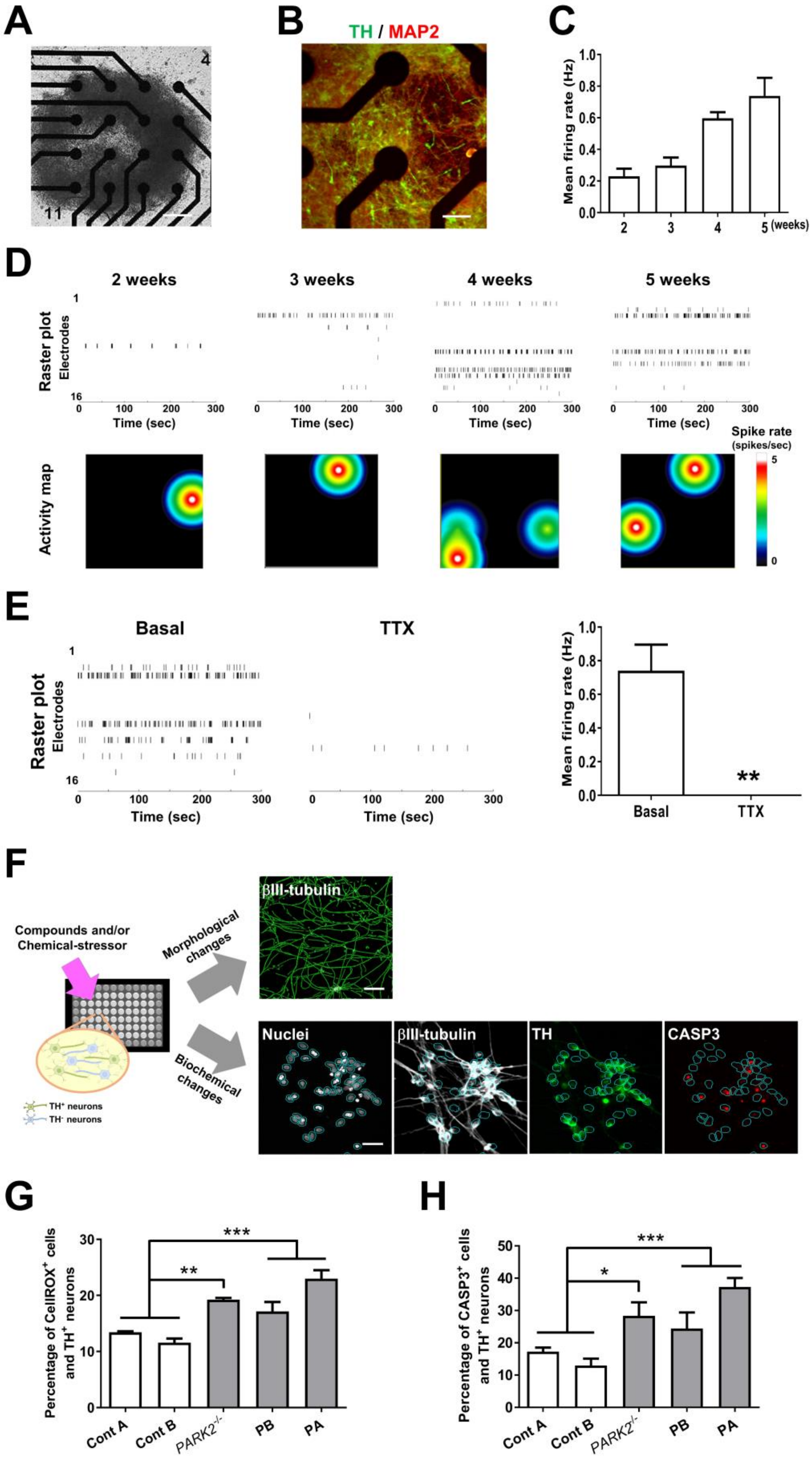

A
B
TH / MAP2
C
Mean firing rate (Hz)
(weeks)
D
2 weeks
3 weeks
4 weeks
5 weeks
Raster plot
Electrodes
Time (sec)
Spike rate
(spikes/sec)
Activity map
E
Basal
TTX
Raster plot
Electrodes
Time (sec)
Mean firing rate (Hz)
**
F
βIII-tubulin
Compounds and/or
Chemical-stressor
Morphological changes
Biochemical changes
TH+ neurons
TH- neurons
Nuclei
βIII-tubulin
TH
CASP3
G
Percentage of CellROX+ cells and TH+ neurons
Cont A
Cont B
PARK2-/-
PB
PA
H
Percentage of CASP3+ cells and TH+ neurons
Cont A
Cont B
PARK2-/-
PB
PA

Figure S2. Characterization of Cultured iPSC-derived Dopaminergic Neurons Using a MEA System and High-Content Image Analysis (Related to Figure 1)

(A and B) Spontaneous firing of cultured iPSC-derived DA neurons (Control B) using a MEA system was determined. Representative images of DA neurons on Day 34. Bright-field image. Scale bar = 300 μm (A). Immunocytochemical staining. Scale bar = 100 μm (B). (C) Quantification of the spontaneous firing rate per active electrode of DA neurons (Control B) using 48-well plate (16 electrodes per well). Data represent the means ± SEM (n = 4 independent biological replicates). (D) Changes in the spontaneous firing pattern of DA neurons (Control B) from 2 to 5 weeks. Representative images of raster plot and activity map in the basal condition. In a raster plot, each row depicts one electrode and every mark represents one spike. (E) The effects of sodium channel blocker tetrodotoxin (TTX) on the mean firing rate. Representative images of raster plot of the DA neurons (Control B) on Day 34 before and after exposure to TTX (10 nM) are shown (left). Quantification is shown (right). Data represent the means ± SEM (n = 4 independent biological replicates). $^{**}p < 0.01$ by an unpaired t-test. (F) Morphological changes were determined by calculating total neurite length per total neuronal cell nucleus. Neurite length of βIII-tubulin^{+} cells was quantified using an algorithm of the IN Cell Investigator (GE Healthcare). For visualization and quantification of apoptotic cells, DA neurons were immunostained with DAPI, βIII-tubulin, TH and CASP3. For cell segmentation, nuclear areas were defined using DAPI staining. Double positive staining of βIII-tubulin and TH was defined as DA neurons. To measure the intracellular oxidative stress of DA neurons, the cells were incubated with CellROX Green. The viability of DA neurons (or intracellular oxidative stress levels) was determined by the proportion of CASP3^{+} (or CellROX Green^{+}) cells among DA neurons. Scale bar = 100 μm (upper) and 20 μm (lower). (G) Elevated intracellular oxidative stress in PARK2- and *PARK2*$^{-/-}$-DA neurons. Intracellular oxidative stress was assessed in DA neurons on Day 14 with an indicator, CellROX. Data represent the means ± SEM (n = 3 independent biological replicates). $^{**}p < 0.01$, $^{***}p < 0.001$ by Dunnett's multiple comparison test. (H) The abundance of apoptotic cells was increased in PARK2- and *PARK2*$^{-/-}$-DA neurons on Day 14. Data represent the means ± SEM (n = 7-10 independent biological replicates). $^{*}p < 0.05$, $^{***}p < 0.001$ by Dunnett's multiple comparison test.

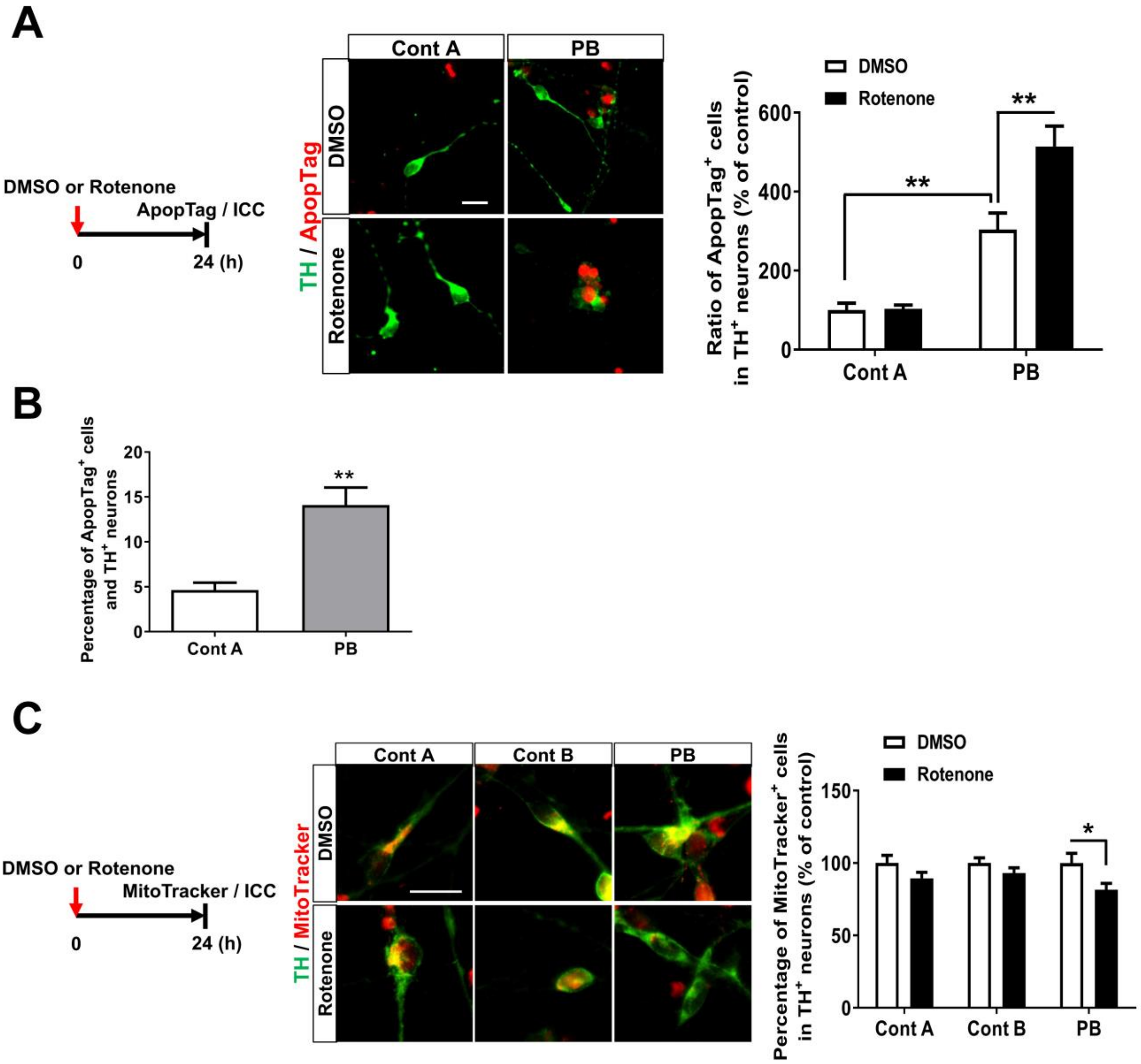
A
DMSO or Rotenone
ApopTag / ICC
0
24 (h)
TH / ApopTag
Cont A
PB
DMSO
Rotenone
Ratio of ApopTag+ cells in TH+ neurons (% of control)
DMSO
Rotenone
**
**
600
400
200
0
Cont A
PB
B
Percentage of ApopTag+ cells and TH+ neurons
20
15
10
5
0
**
Cont A
PB
C
DMSO or Rotenone
MitoTracker / ICC
0
24 (h)
TH / MitoTracker
Cont A
Cont B
PB
DMSO
Rotenone
Percentage of MitoTracker+ cells in TH+ neurons (% of control)
DMSO
Rotenone
150
100
50
0
*
Cont A
Cont B
PB

Figure S3. PARK2 iPSC-derived Dopaminergic Neurons Showed Increased Susceptibility to Rotenone-induced Stress (Related to Figures 1 and 2)

(A) Immunocytochemical analysis of ApopTag^{+} cells in the control (Control A) and PARK2 (PB)-DA neurons on Day 14 with or without rotenone treatment (10 μM, 24 h) using the TUNEL method. Representative staining images of ApopTag^{+} cells are shown (middle). Quantification of apoptotic cells in TH^{+} neurons is shown (right). Data represent the means ± SEM (n = 3-6 independent biological replicates). **$p < 0.01$ by Tukey's multiple comparison test. Scale bar = 20 μm. (B) The abundance of apoptotic cells was increased in PARK2 (PB)-DA neurons on Day 14. Data represent the means ± SEM (n = 6 independent biological replicates). **$p < 0.01$ by an unpaired t-test. (C) Reduced the MitoTracker^{+} fraction by rotenone treatment in PARK2(PB)-DA neurons on Day 14 compared with the control (Control A and B). Representative staining images of MitoTracker^{+} cells with or without rotenone exposure (10 μM, 24 h) are shown (middle). Quantification of MitoTracker^{+} cells is shown (right). Data represent the means ± SEM (n = 3-6 independent biological replicates). *$p < 0.05$ by a t-test with Sidak's correction. Scale bar = 20 μm.

A

Compound	Description	Selectivity of Ca^{2+} channels
Benidipine	a CCA	L, N, T
Cinnarizine	a CCA and antihistamine	L, T
Amiodarone	a CCA, antianginal and antiarrhythmic agent	L, T
Suloctidil	a CCA	Non-selective
Proscillaridin	a cardiac glycoside	-
Tyrothricin	an antibiotic	-
Bacampicillin	an antibiotic	-
Ivermectin	a antiparasite medication	-
Chlorhexidine	a disinfectant and topical anti-infective agent	-

CCA, Ca^{2+} channel antagonist

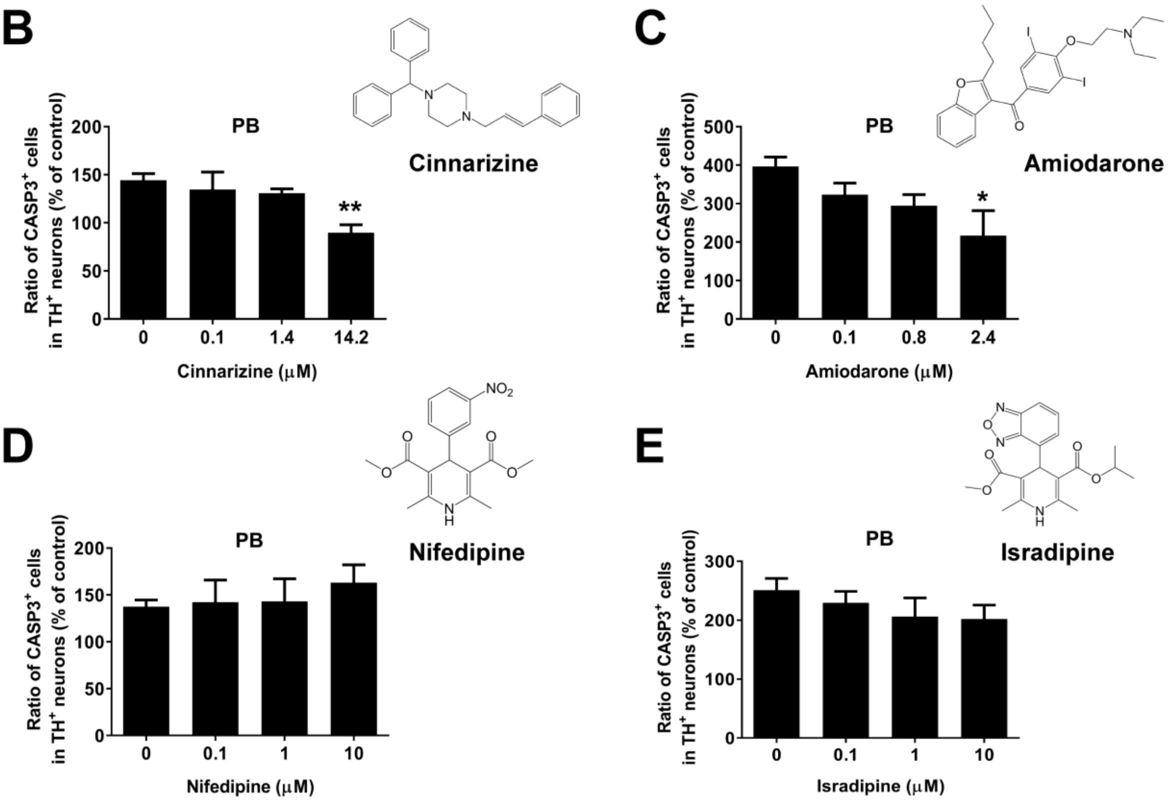

Figure S4. Categories of 9 Compounds Confirmed as Hits by a Concentration Dependency Assay (Related to Figure 3)

(A) The compounds were categorized based on their potential effects. (B-E) Protective effects of cinnarizine, amiodarone, nifedipine and isradipine on rotenone-mediated (10 μM, 24 h) apoptosis. Immunocytochemical analysis of $CASP3^{+}$ cells in PARK2 (PB)-DA neurons was conducted with cinnarizine (B), amiodarone (C), nifedipine (D) and isradipine (E). Data represent the means ± SEM (n = 3-12 independent biological replicates). $^{*}p < 0.05$, $^{**}p < 0.01$ by Dunnett's multiple comparison test.

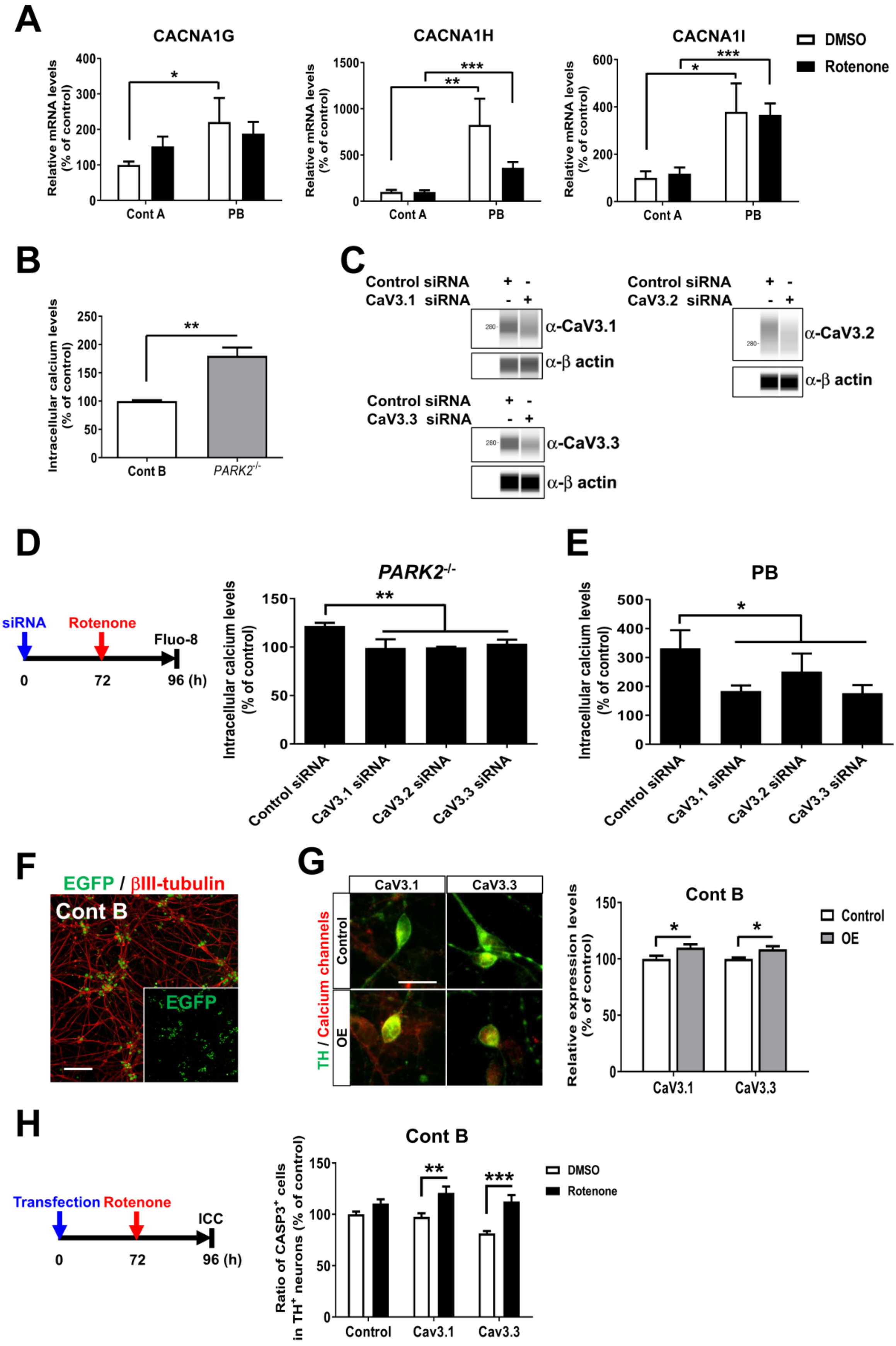
A
CACNA1G
CACNA1H
CACNA1I
DMSO
Rotenone
Relative mRNA levels (% of control)
Cont A
PB
B
Intracellular calcium levels (% of control)
Cont B
PARK2-/-
C
Control siRNA
CaV3.1 siRNA
α-CaV3.1
α-β actin
CaV3.2 siRNA
α-CaV3.2
CaV3.3 siRNA
α-CaV3.3
D
siRNA
Rotenone
Fluo-8
0
72
96 (h)
PARK2-/-
Control siRNA
CaV3.1 siRNA
CaV3.2 siRNA
CaV3.3 siRNA
E
PB
F
EGFP / βIII-tubulin
Cont B
EGFP
G
CaV3.1
CaV3.3
Control
OE
TH / Calcium channels
Relative expression levels (% of control)
H
Transfection
Rotenone
ICC
Ratio of CASP3+ cells in TH+ neurons (% of control)
Control
Cav3.1
Cav3.3

Figure S5. Dysregulation of Calcium Homeostasis in Dopaminergic Neurons by T-type Calcium Channels Contributed to Disease-related Phenotypes of PD (Related to Figure 5)

(A) The qRT-PCR reaction was performed on Day 14 for the T-type calcium channel subtypes (CACNA1G (CaV3.1), CACNA1H (CaV3.2) and CACNA1I (CaV3.3)). Data represent the means ± SEM (n = 4-8 independent biological replicates). $*p < 0.05$, $**p < 0.01$, $***p < 0.001$ by an unpaired t-test. (B) Measurement of intracellular calcium levels of neurons (Control B and *PARK2*$^{-/-}$) on Day 14 by the fluorescent calcium indicator Fluo-8 AM. Data represent the means ± SEM (n = 4 independent biological replicates). $**p < 0.01$ by an unpaired t-test.

(C) Expression of T-type calcium channel subtypes after siRNA transfection was examined using subtype specific antibodies. An antibody against β actin is used as an internal control. (D and E) The involvement of T-type calcium channel subtypes in the regulation of intracellular calcium levels was examined in the *PARK2*$^{-/-}$- and PARK2 (PB)-derived DA neurons on Day 14 with rotenone treatment (10 μM, 24 h). The cells were transfected with siRNA against the various T-type calcium channel subtypes for 72 h prior to rotenone treatment. Data represent the means ± SEM (n = 3 independent biological replicates). $*p < 0.05$, $**p < 0.01$ by an unpaired t-test. (F) Representative image of EGFP^{+} cells in βIII-tubulin^{+} neurons after 72 h of transfection with the CMV-EGFP plasmid in DA neurons (Control B) using Lipofectamine 3000. After transfection, immunocytochemical staining was performed with antibody for βIII-tubulin. Insets are the zoomed-out images of EGFP^{+} signals. Scale bar = 100 μm. (G) Immunocytochemistry was performed in DA neurons (Control B) after overexpression (OE) of the T-type calcium channel subtypes (CaV3.1 or CaV3.3). Representative image of calcium channel subtypes in DA neurons (left). The relative expression levels of calcium channel subtypes are shown (right). Data represent the means ± SEM (n = 6 independent biological replicates). $*p < 0.05$ by a t-test with Sidak's correction. (H) Immunocytochemical analysis of CASP3^{+} cells in DA neurons (Control B) transfected for 72 h with the CMV-T-type calcium channel subtypes (CaV3.1 or CaV3.3) plasmid on Day 14 with or without rotenone exposure (10 μM, 24 h). Data represent the means ± SEM (n = 6 independent biological replicates). $**p < 0.01$, $***p < 0.001$ by Tukey's multiple comparison test.

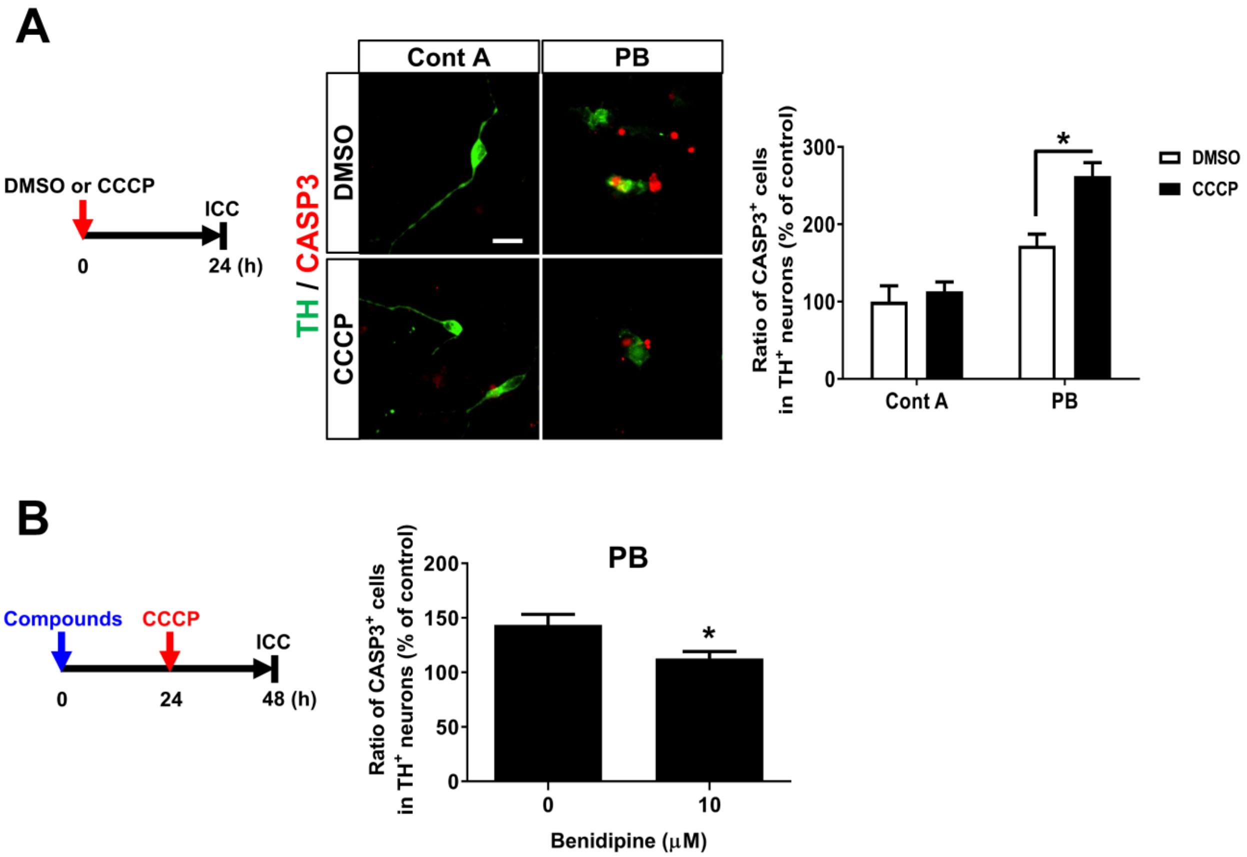
A
DMSO or CCCP
ICC
0
24 (h)
Cont A
PB
DMSO
CCCP
TH / CASP3
Ratio of CASP3+ cells in TH+ neurons (% of control)
300
200
100
0
Cont A
PB
*
DMSO
CCCP
B
Compounds
CCCP
ICC
0
24
48 (h)
PB
Ratio of CASP3+ cells in TH+ neurons (% of control)
200
150
100
50
0
*
0
10
Benidipine (μM)

Figure S6. Calcium Channel Antagonist Protected against Mitochondrial Oxidative Phosphorylation Uncoupler-induced Apoptosis in PARK2 iPSC-derived Dopaminergic Neurons (Related to Figures 2, 3 and 6)

(A) Immunocytochemical analysis of CASP3^{+} cells in the control (Control A) and PARK2 (PB)-DA neurons on Day 14 with or without carbonyl cyanide m-chlorophenylhydrazone (CCCP) treatment (10 µM, 24 h). Representative images of CASP3 immunostaining are shown (left). Scale bar = 20 µm. Quantification is shown (middle). Data represent the means ± SEM (n = 3 independent biological replicates). *$p < 0.05$ by Tukey's multiple comparison test. (B) Protective effects of benidipine on CCCP-mediated (10 µM, 24 h) apoptosis. Data represent the means ± SEM (n = 6 independent biological replicates). *$p < 0.05$ by an unpaired t-test.

Table S1. Antibody list used in this study

Antibody	Dilution	Host	Catalogue number	Supplier	Location
Primary antibodies;					
anti-SOX1	1:1000	Goat	AF3369	R&D Systems	Minneapolis, MN
anti-SOX2	1:500	Mouse	MAB2018	R&D Systems	Minneapolis, MN
anti-DACH1	1:200	Rabbit	10914-1-AP	Proteintech	Rosemont, IL
anti-nestin	1:500	Mouse	MAB5326	Merck Millipore	Billerica, MA
anti-βIII-tubulin	1:1000	Mouse	T8660	Sigma-Aldrich	St. Louis, MO
anti-MAP2	1:500	Rabbit	AB5622	Merck Millipore	Billerica, MA
anti-TH	1:1000	Sheep	AB1542	Merck Millipore	Billerica, MA
anti-TH	1:500	Rabbit	AB152	Merck Millipore	Billerica, MA
anti-GFAP	1:500	Rat	13-0300	Thermo Fisher Scientific	Waltham, MA
anti-FOXA2	1:200	Mouse	H00003170-M01	Abnova	Taipei, Taiwan
anti-EN1	1:500	Mouse	4G11	Developmental Studies Hybridoma Bank	Iowa, IA
anti-NURR1	1:500	Mouse	PP-N1404-00	R&D Systems	Minneapolis, MN
anti-GIRK2	1:400	Rabbit	APC-006	Alomone Laboratories	Jerusalem, Israel
anti-cleaved caspase-3	1:1000	Rabbit	9661	Cell Signaling Technology	Danvers, MA
anti-CaV3.1	1:200	Rabbit	ACC-021	Alomone Laboratories	Jerusalem, Israel
anti-CaV3.2	1:200	Rabbit	ACC-025	Alomone Laboratories	Jerusalem, Israel
anti-CaV3.3	1:200	Rabbit	ACC-009	Alomone Laboratories	Jerusalem, Israel
Secondary antibodies;					
Alexa Fluor 488-conjugated anti-sheep IgG	1:500	Donkey	A11015	Thermo Fisher Scientific	Waltham, MA
Alexa Fluor 568-conjugated anti-sheep IgG	1:500	Donkey	A21099	Thermo Fisher Scientific	Waltham, MA
Cy2-conjugated anti-rabbit IgG	1:500	Donkey	711-225-152	Jackson Immuno Research	West Grove, PA
Cy2-conjugated anti-mouse IgG	1:500	Donkey	715-225-150	Jackson Immuno Research	West Grove, PA
Cy3-conjugated anti-goat IgG	1:500	Donkey	705-165-147	Jackson Immuno Research	West Grove, PA
Cy3-conjugated anti-mouse IgG	1:500	Donkey	715-165-150	Jackson Immuno Research	West Grove, PA
Cy3-conjugated anti-rat IgG	1:500	Donkey	712-165-153	Jackson Immuno Research	West Grove, PA
Cy3-conjugated anti-rabbit IgG	1:500	Donkey	711-165-152	Jackson Immuno Research	West Grove, PA
Cy5-conjugated anti-mouse IgG	1:500	Donkey	715-175-151	Jackson Immuno Research	West Grove, PA

DACH1, dachshund homolog 1; MAP2, microtuble-associated protein 2; TH, tyrosine hydroxylase; GFAP, glial fibrillary acidic protein; FOXA2, forkhead box protein A2; EN1, engrailed-1; NURR1, nur-related factor 1; GIRK2, G-protein-activated inwqrd rectifier potassium channel 2

Table S2. Compound list used in this study

Compound	Molecular weight	Catalogue number	Supplier	Location
Benidipine	542.02	B6813	Sigma-Aldrich	St. Louis, MO
Cinnarizine	368.51	C5270	Sigma-Aldrich	St. Louis, MO
Amiodarone	681.77	A8423	Sigma-Aldrich	St. Louis, MO
Suloctidil	337.56	S9384	Sigma-Aldrich	St. Louis, MO
Proscillaridin	530.65	P2428	Sigma-Aldrich	St. Louis, MO
Tyrothricin	1228.44	T3000000	Sigma-Aldrich	St. Louis, MO
Bacampicillin	501.98	1047300	U. S. Pharmacopeia	Rockville, MD
Ivermectin	875.09	090-05521	Wako	Osaka, Japan
Chlorhexidine	578.40	220557	Calbiochem	San Diego, CA
Nifedipine	346.33	N7634	Sigma-Aldrich	St. Louis, MO
Isradipine	371.39	I6658	Sigma-Aldrich	St. Louis, MO
ML218	369.33	SML0385	Sigma-Aldrich	St. Louis, MO
Rotenone	394.42	R8875	Sigma-Aldrich	St. Louis, MO
TTX	319.27	4368-28-9	Wako	Osaka, Japan
CCCP	204.62	C2759	Sigma-Aldrich	St. Louis, MO
DMSO	78.13	D5879	Sigma-Aldrich	St. Louis, MO

DMSO, dimethyl sulfoxide; TTX, tetrodotoxin; CCCP, carbonyl cyanide m-chlorophenylhydrazone

Supplemental Experimental Procedures

Generation of Patient-Specific and Isogenic iPSC-derived Neural Progenitor Cells

The iPSC line 201B7 (Takahashi et al., 2007) (a gift from Dr. Shinya Yamanaka), PARK2 iPSC lines PA9 and PB2 (Imaizumi et al., 2012) and PARK6 iPSC line PKB3 (Shiba-Fukushima et al., 2017) were established and maintained as previously described. A PARK2-deficient iPSC line (B7PA21) was generated by applying CRISPR-Cas9 to 201B7 cells (Figure S1) (Suda et al., 2018). In brief, a targeting donor DNA plasmid (pUC- 5'3'PARK2-PurTK) was used to disrupt exon 2 of *PARK2* gene by homologous recombination. The CSIV-U6-*PARK2* (Ex2)-sgRNA-L&R-EF-Csy4-2A-Cas9 was used as a house-made all-in-one vector. The 201B7 was suspended in Opti-MEM (Thermo Fisher Scientific, Waltham, MA) containing Y-27632 (Wako, Tokyo, Japan), house-made all-in-one vector and targeting donor DNA vector plasmid. Electroporation of plasmid DNA was performed using a NEPA21 electroporator (Nepa Gene Co., Ichikawa, Japan). As shown in Figure S1B, homologous recombination in the *PARK2* gene was confirmed by Southern blotting analysis. Furthermore, the expression levels of Parkin was determined by the capillary electrophoresis immunoassay (Figure S1C). The iPSC-derived NPC cell line AF22 (Falk et al., 2012) was kindly provided by Dr. Austin Smith. NPCs were established from the 201B7, PA9, PB2, PKB3, and B7PA21 iPSC lines as previously described (Falk et al., 2012).

Cell Culture

iPSC-derived NPC cultures were maintained in N2 media supplemented with 10 ng/mL epidermal growth factor (EGF; Peprotech, Rocky Hill, NJ) and 10 ng/mL basic fibroblast growth factor (bFGF; StemCultures, Rensselaer, NY) as previously described (Falk et al., 2012). To generate midbrain DA neurons, we cultured the NPCs in N2 media supplemented with 200 ng/mL FGF8 (Peprotech) and 200 ng/mL sonic hedgehog (SHH; R&D Systems, Minneapolis, MN) for 7 days and then further cultured them for 7 days with FGF8, SHH and 1 μM CHIR99021 (CHIR; Stemgent, Cambridge, MA) in Matrigel (BD Biosciences, Franklin Lakes, NJ)-coated dishes. To consistently induce DA neurons, we cryopreserved cells that had been pretreated with FGF8 and SHH for 7 days, using Cell Banker-2 (ZENOAQ, Fukushima, Japan). After midbrain patterning treatment the cells were allowed to differentiate into DA neurons in KBM Neural Stem Cell media (KohjinBio, Saitama, Japan) supplemented with B27 (1:50; Thermo Fisher Scientific)

(referred to as MHM media) along with 20 ng/mL brain-derived neurotrophic factor (BDNF; R&D Systems), 20 ng/mL glial-derived neurotrophic factor (GDNF; Peprotech), 1 ng/mL transforming growth factor-β3 (TGF-β3; R&D Systems), 200 μM ascorbic acid (A.A.; Sigma-Aldrich, St. Louis, MO), 500 μM dibutyryl-cAMP (db-cAMP; Sigma-Aldrich) and 2 μM DAPT (Sigma-Aldrich) (referred to as differentiation media) and seeded at a density of 50,000 cells/cm^2 on 96-well plates coated with poly-D-lysine (PDL; Greiner Bio-One, Kremsmünster, Austria) and 10 μg/mL laminin (Thermo Fisher Scientific). The medium was changed every 3 or 4 days.

Measurement of Dopamine Release

Dopamine release was quantified using a Dopamine Research ELISA Kit (Labor Diagnostika Nord, Nordhorn, Germany) in samples cultured for 3 days at each stage of differentiation, such as 2, 3, and 4 weeks (Figures 1E and 1F). The cells at the last step of differentiation into DA neurons were plated at a density of 200,000 cells/cm^2. The assay was performed following the manufacturer's instructions. Absorbance was read using an iMark microplate reader (BIO-RAD, Hercules, CA) set to 450 nm with a reference wavelength of 595 nm.

Microelectrode Array Recording

A microelectrode array (MEA) recording was performed using the Maestro system (Axion Biosystems, Atlanta, GA) as previously described (Isoda et al., 2016). To prepare iPSC-derived DA neurons for array recording, midbrain patterning treated cells were seeded at a density of 100,000 cells/well on 48-well plates (CytoView MEA) coated with 100 μg/mL PDL (Sigma-Aldrich) and 10 μg/mL laminin (Figures S2A-S2E). A detection threshold was set to +6.0× standard deviation of the baseline electrode noise. Spike raster plots were analyzed using a Neural Metric Tool (Axion Biosystems). The active electrode criterion was defined as an electrode having an average of more than 5 spikes/min. To ascertain the biological nature of the recordings, the sodium channel blocker tetrodotoxin (Wako) was added to the medium, and neuronal activity was recorded (Figure S2E) (Du et al., 2016).

Immunocytochemistry

Immunocytochemistry was performed as previously described (Tabata et al., 2015). The primary antibodies and secondary antibodies used in this study are listed in Table S1. Dopamine was detected using a STAINperfect Immunostaining Kit (rabbit anti-dopamine; 1:500; ImmuSmol, Pessac, France) (Monzel et al., 2017) according to the manufacturer's protocol.

High-Content Analysis

Images were obtained using an IN Cell Analyzer 6000 (GE Healthcare, Chicago, IL). A set of 20 fields per well were scanned at 20× magnification, and image analysis was conducted with the IN Cell Developer Toolbox 1.9 software (GE Healthcare) using custom-developed analysis protocols. For cell segmentation and the nucleus counts, images were acquired using DAPI staining (Figures S2F-S2H). In the neural cell population analysis, we divided the number of cells positive for each marker by the total number of cells (stained with DAPI). The neurite arborization of βIII-tubulin^{+} neurons was quantified using an algorithm in the IN Cell Developer Toolbox 1.9. Morphological changes were determined by calculating the total neurite length per total neuronal cell. For the visualization and quantification of intracellular oxidative stress or mitochondria, the cells were incubated with CellROX Green (1 μM, Thermo Fisher Scientific) or MitoTracker Red CMXRos (0.5 μM, Thermo Fisher Scientific) for 30 min at 37°C and 5% CO_2 incubated with MHM media, respectively. Thereafter, immunostaining for βIII-tubulin and TH was performed. The intracellular oxidative stress levels or mitochondria were determined by the proportion of CellROX^{+} cells or MitoTracker^{+} cells (Figure S3C) among DA neurons. To measure the viability of DA neurons, we quantified apoptotic cells by immunohistochemistry for CASP3. In addition, we used an ApopTag Fluorescein In Situ Apoptosis Detection Kit (sheep anti-digoxigenin fluorescein; Merck Millipore, Billerica, MA) based on the terminal deoxynucleotidyl transferase dUTP nick end labeling (TUNEL) method according to the manufacturer's protocol as an alternative method to examine apoptosis (Figures S3A and S3B).

Compound Screening

False positive compounds among the primary hit compounds were excluded by concentration-dependency assays. The results of the primary screening were made into a dot plot by the software TIBCO Spotfire (TIBCO Software, Palo Alto, CA).

Calcium Imaging

The cells were incubated with a fluorescent calcium indicator Fluo-8 acetoxymethyl (AM; 5 µM, AAT Bioquest, Sunnyvale, CA) in Hank's Balanced Salt Solution (Nacalai Tesque) with Pluronic F-127 (0.02%; Dojindo, Kumamoto, Japan) and Hoechst-33342 (2 µg /mL; Sigma-Aldrich) for 30 min at 37°C and 5% CO_2. To assess the baseline calcium levels, we immediately scanned the cells using an IN Cell Analyzer 6000. A set of 4 fields per well were scanned at 10× magnification. Subsequently, the cells were incubated with a calcium ionophore, A23187 (4 µM; Tocris Bioscience, Bristol, UK), for 30 min and further incubated with a calcium-selective chelating agent, EGTA (10 mM; Dojindo), for 30 min. The intracellular calcium concentration was calculated using the following equation:

Intracellular calcium level = K_D [$(R - R_{min}) / (R_{max} - R)$]

The dissociation constant (K_D) value was 389 nM, as described in the manufacturer's protocol. The R_{min} value was obtained in the presence of both A23187 and EGTA. The R_{max} was obtained in the presence of A23187 only. For cell segmentation and nucleus counts, images were acquired using Hoechst-33342 staining.

Gene Silencing with siRNA

DA neurons were incubated for 72 h with Accell siRNA (1 µM, Thermo Fisher Scientific) in a 50:50 mix of Accell delivery media and differentiation media. The Accell siRNAs were Accell Non-targeting siRNA #1 (#D-001910-10), CaV3.1 (#E-006127-00), CaV3.2 (#E-006128-00), and CaV3.3 (#E-006129-00) (Figure 5D). A quantitative reverse transcription polymerase chain reaction (qRT-PCR) was performed to measure the level of gene expression 72 h after the transfection using the Cells to CT Kit (Thermo Fisher Scientific). The qRT-PCR reaction was performed in duplicate using TaqMan gene expression assays (Thermo Fisher Scientific) for GAPDH (Hs02758991_g1), CaV3.1 (Hs00367969_m1), CaV3.2 (Hs01103527_m1), and CaV3.3 (Hs01096207_m1) on a ViiA 7 real-time PCR system (Thermo Fisher Scientific) (Figure S5A). Furthermore, the expression level of the T-type calcium channel subtypes was determined by a capillary electrophoresis immunoassay (Figure S5C).

Plasmid DNA Transfection

The plasmid pEGFP-N1 was purchased from Clontech Laboratories (now Takara Bio USA, Mountain View, CA). The hα1Ga (CACNA1G)–pDsRed and a1Ic (CACNA1I)-HE3-pcDNA3 expression plasmid DNA were obtained from Addgene (#45811 and #45810, respectively; Cambridge, MA). Transfection of the plasmid into iPSC-derived DA neurons was performed using Lipofectamine 3000 transfection reagent (Thermo Fisher Scientific) according to the manufacturer's protocol. Immunocytochemistry was performed to measure the level of expression 72 h after the transfection (Figures S5F-S5H).

Chemicals

All chemicals used in this study are listed in Table S2. The chemical structures were drawn using ChemDraw Prime 16.0 (PerkinElmer, Waltham, MA).

Supplemental References

Du, F., Yu, Q., Shijun, Y., Doris, C., and Yan, S.S. (2016). Development and Dynamic Regulation of Mitochondrial Network in Human Midbrain Dopaminergic Neurons Differentiated from iPSCs. Stem Cell Reports. *7*, 678-692.

Falk, A., Koch, P., Kesavan, J., Takashima, Y., Ladewig, J., Alexander, M., Wiskow, O., Tailor, J., Trotter, M., Pollard, S., et al. (2012). Capture of neuroepithelial-like stem cells from pluripotent stem cells provides a versatile system for in vitro production of human neurons. PLoS One. *7*, e29597.

Imaizumi, Y., Okada, Y., Akamatsu, W., Koike, M., Kuzumaki, N., Hayakawa, H., Nihira, T., Kobayashi, T., Ohyama, M., Sato, S., et al. (2012). Mitochondrial dysfunction associated with increased oxidative stress and α-synuclein accumulation in PARK2 iPSC-derived neurons and postmortem brain tissue. Molecular Brain. *5*, 35.

Monzel, A.S., Smits, L.M., Hemmer, K., Hachi, S., Moreno, E.L., van Wuellen, T., Jarazo, J., Walter, J., Brüggemann, I., Boussaad, I., et al. (2017). Derivation of human midbrain-specific organoids from neuroepithelial stem cells. Stem Cell Reports. *8*, 1144-1154.

Shiba-Fukushima, K., Ishikawa, K.I., Inoshita, T., Izawa, N., Takanashi, M., Sato, S., Onodera, O., Akamatsu, W., Okano, H., Imai, Y., et al. (2017). Evidence that phosphorylated ubiquitin signaling is involved in the etiology of Parkinson's disease. Hum Mol Genet.

Suda, Y., Kuzumaki, N., Sone, T., Narita, M., Tanaka, K., Hamada, Y., Iwasawa, C., Shibasaki, M., Maekawa, A., Matsuo, M., et al. (2018). Down-regulation of ghrelin receptors on dopaminergic neurons in the substantia nigra contributes to Parkinson's disease-like motor dysfunction. Mol Brain. *11*, 6.

Tabata, Y., Murai, N., Sasaki, T., Taniguchi, S., Suzuki, S., Yamazaki, K., and Ito, M. (2015). Multiparametric phenotypic screening system for profiling bioactive compounds using human fetal hippocampal neural stem/progenitor cells. Journal of Biomolecular Screening. *20*, 1074-1083.

Takahashi, K., Tanabe, K., Ohnuki, M., Narita, M., Ichisaka, T., Tomoda, K., and Yamanaka, S. (2007). Induction of pluripotent stem cells from adult human fibroblasts by defined factor. Cell. *131*, 861-872.
