# Supplementary material for: T-type Calcium Channels Determine the Vulnerability of Dopaminergic Neurons to Mitochondrial Stress in Familial Parkinson Disease
Source: Stem Cell Reports. 2018 Oct 18;11(5):1171–84. doi: 10.1016/j.stemcr.2018.09.006 (PMC6234903; doi:10.1016/j.stemcr.2018.09.006)
Supplement: Document S2. Article plus Supplemental Information [file mmc2.pdf]

Stem Cell Reports
Article
OPEN ACCESS

T-type Calcium Channels Determine the Vulnerability of Dopaminergic Neurons to Mitochondrial Stress in Familial Parkinson Disease

Yoshikuni Tabata,[1,2] Yoichi Imaizumi,[2] Michiko Sugawara,[2] Tomoko Andoh-Noda,[1] Satoe Banno,[1] MuhChyi Chai,[1] Takefumi Sone,[1] Kazuto Yamazaki,[2] Masashi Ito,[2] Kappei Tsukahara,[2] Hideyuki Saya,[3] Nobutaka Hattori,[4] Jun Kohyama,[1,*] and Hideyuki Okano[1,*]

[1]Department of Physiology, Keio University School of Medicine, 35 Shinanomachi, Shinjuku-ku, Tokyo 160-8582, Japan
[2]Tsukuba Research Laboratories, Eisai Co., Ltd, 5-1-3 Tokodai, Tsukuba-shi, Ibaraki 300-2635, Japan
[3]Division of Gene Regulation, Institute for Advanced Medical Research, Keio University School of Medicine, 35 Shinanomachi, Shinjuku-ku, Tokyo 160-8582, Japan
[4]Department of Neurology, Juntendo University Graduate School of Medicine, Tokyo 113-8421, Japan
*Correspondence: jkohyama@a7.keio.jp (J.K.), hidokano@a2.keio.jp (H.O.)
https://doi.org/10.1016/j.stemcr.2018.09.006

SUMMARY

Parkinson disease (PD) is a progressive neurological disease caused by selective degeneration of dopaminergic (DA) neurons in the substantia nigra. Although most cases of PD are sporadic cases, familial PD provides a versatile research model for basic mechanistic insights into the pathogenesis of PD. In this study, we generated DA neurons from PARK2 patient-specific, isogenic *PARK2* null and PARK6 patient-specific induced pluripotent stem cells and found that these neurons exhibited more apoptosis and greater susceptibility to rotenone-induced mitochondrial stress. From phenotypic screening with an FDA-approved drug library, one voltage-gated calcium channel antagonist, benidipine, was found to suppress rotenone-induced apoptosis. Furthermore, we demonstrated the dysregulation of calcium homeostasis and increased susceptibility to rotenone-induced stress in PD, which is prevented by T-type calcium channel knockdown or antagonists. These findings suggest that calcium homeostasis in DA neurons might be a useful target for developing new drugs for PD patients.

INTRODUCTION

Parkinson disease (PD) is one of the most common neurodegenerative disorders, and PD patients display progressive motor dysfunction such as tremor, bradykinesia, rigidity and postural instability due to a preferential loss of dopaminergic (DA) neurons in the substantia nigra (SN) (Damier et al., 1999). While the majority of PD cases are sporadic, approximately 10% of cases (Verstraeten et al., 2015) are explained by the dysregulation of proteins including PARKIN (PARK2) (Kitada et al., 1998; Mizuno et al., 2008), PTEN-induced putative kinase 1 (PINK1 or PARK6) (Valente et al., 2004), α-synuclein (PARK1/4) (Polymeropoulos et al., 1997), and leucine-rich repeat serine/threonine protein kinase 2 (LRRK2 or PARK8) (Funayama et al., 2002; Zimprich et al., 2004). These forms of familial PD have been providing important insights into the pathogenesis of PD and have opened up new areas of investigation. For example, PD-related genes such as *PINK1* (*PARK6*) and *PARKIN* (*PARK2*) are involved in mitochondrial homeostasis and stress responses (Du et al., 2017; Kalia and Lang, 2015; Narendra et al., 2012). PARK1/4 is involved in α-synuclein accumulation, which is the pathological feature of PD (Kalia and Lang, 2015), and PARK8 has also been reported to be associated with impaired autophagy. However, the findings obtained from cellular models and animal models do not always accurately reflect the pathogenesis of PD patients due to different cellular contexts or vulnerability to disease-relevant mutations (Blesa and Przedborski, 2014). Therefore, despite these advances, no effective therapeutic treatments have been developed.

The recent progress of human induced pluripotent stem cell (iPSC)-assisted technology presents new opportunities for disease modeling based on precise cellular contexts, which had previously been difficult to obtain, especially in neurodegenerative disorders (Okano and Yamanaka, 2014). In the case of PD, patient-derived iPSCs would be a direct source for midbrain DA neurons, a disease-relevant cell type. Accordingly, there has been a series of studies demonstrating disease-relevant phenotypes using PD-derived iPSCs (Drouin-Ouellet and Barker, 2012). Although these studies have elegantly demonstrated disease-related phenotypes, a more efficient protocol to generate DA neurons from patient-derived iPSCs would lead to the identification of mechanisms whereby PD patients develop DA-neuron-specific phenotypes.

Here, we developed an efficient directed differentiation protocol to generate DA neurons with less variation of efficacy than previous protocols (Imaizumi et al., 2012; Ren et al., 2015; Shaltouki et al., 2015). We used PARK2 patient-specific and isogenic *PARK2* null (*PARK2*$^{-/-}$) iPSC-derived DA neurons (PARK2-DA neurons and *PARK2*$^{-/-}$-DA neurons, respectively) for *in vitro* disease modeling and found that these neurons exhibited neurite abnormalities, elevated oxidative stress, and apoptosis. We also performed a phenotypic screening to identify neuroprotective compounds and identified benidipine, a voltage-gated calcium channel antagonist, as a potential chemical targeting

This is an open access article under the CC BY license (http://creativecommons.org/licenses/by/4.0/).

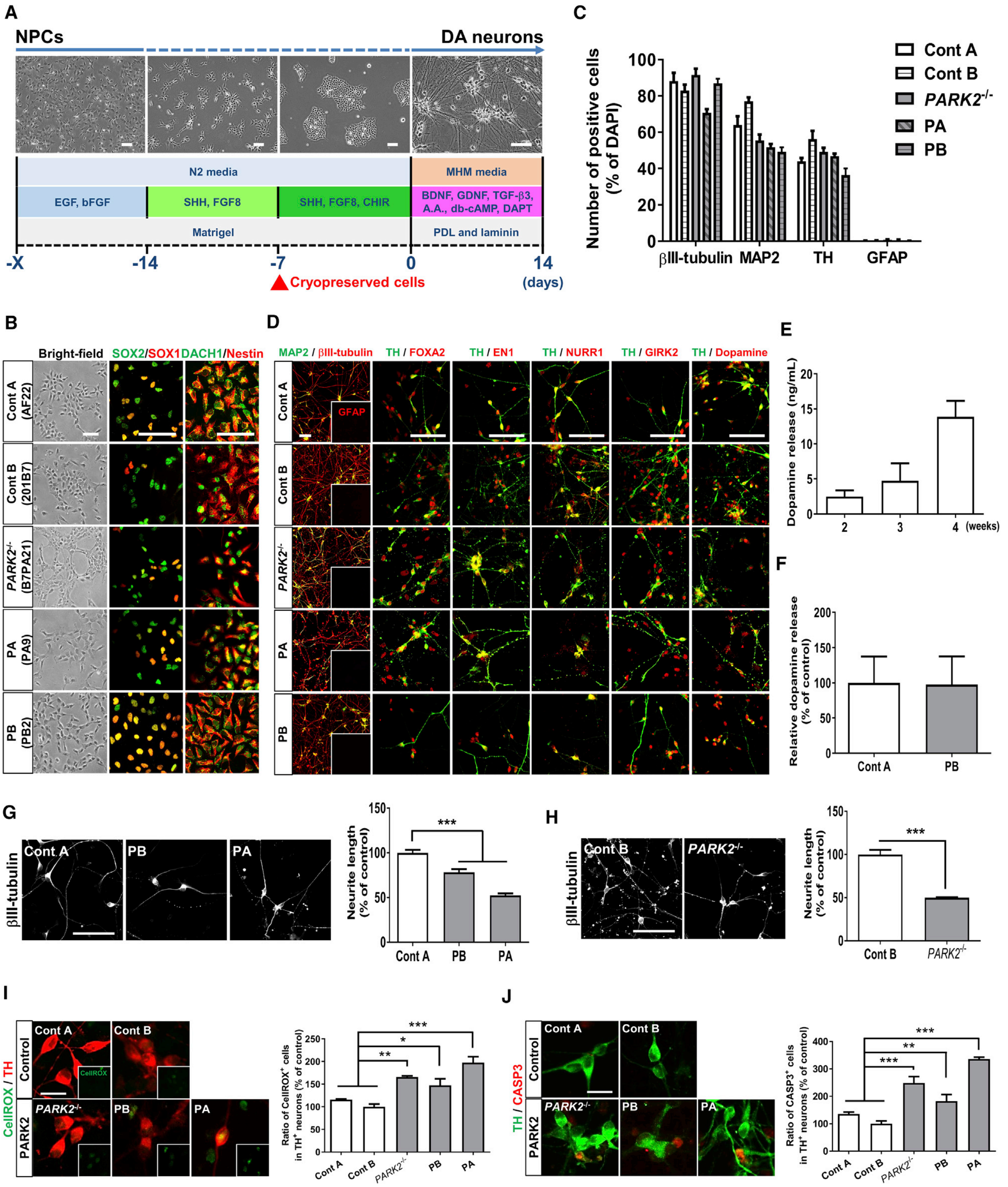

Figure 1. Characterization of Dopaminergic Neurons Derived from PARK2 Patient-Specific and Isogenic *PARK2*$^{-/-}$ iPSC Lines

(A) Experimental paradigm for differentiation of human iPSC-derived NPCs toward DA neurons. Scale bars, 100 μm.

(B) Properties of iPSC-derived NPCs from the Control A (AF22), Control B (201B7), *PARK2*$^{-/-}$ (B7PA21), PA (PA9), and PB (PB2) lines. Immunocytochemical staining was performed with antibodies against neural stem cell-associated markers (SOX1, SOX2, DACH1, and nestin). Scale bar, 100 μm.

(legend continued on next page)

PD. Importantly, we found that the selective vulnerability of DA neurons to rotenone-induced stress in PARK2 was attributable to the dysregulation of intracellular calcium homeostasis via T-type calcium channels. In summary, we have established a robust platform to model PD in a dish and revealed an additional layer of the pathogenesis of PD, offering a potential therapeutic target.

RESULTS

Characterization of Dopaminergic Neurons Derived from PARK2 Patient-Specific and Isogenic *PARK2*$^{-/-}$ iPSC Lines

In an attempt to identify the chemical compounds for PD, we developed an efficient directed differentiation protocol to establish an *in vitro* disease model using PD patient-specific iPSC-derived DA neurons (Figure 1A). As a feature of this protocol, it is possible to use cryopreserved neural progenitor cells (NPCs), which enables us to perform stable differentiation induction, reproducible disease phenotypic analysis, and compound screening in a large number of cells with a uniform frozen cell lot. As an entry point, NPCs were generated from the iPSCs established from two PARK2 patients, PA and PB (Figure 1B). For comparison, NPCs derived from control iPSCs were also used (Figure 1B). In addition, iPSC-NPCs derived from a *PARK2*$^{-/-}$ iPSC line (B7PA21) were also generated to evaluate the disease-relevant phenotypes in the same genetic background (Figures 1B and S1). We initially examined the cellular properties after the differentiation of iPSC-NPCs toward midbrain DA neurons. As shown in Figure 1C, the efficiency of neuronal differentiation was assessed on differentiation day 14 by the number of cells positive for neuronal markers including βIII-tubulin and microtubule-associated protein 2 (MAP2); βIII-tubulin^{+} neurons constituted approximately 84% of the total cells among all the lines. In our protocol, there were very few glial fibrillary acidic protein (GFAP)$^{+}$ cells, indicating that astrocytic differentiation was negligible. Importantly, tyrosine hydroxylase (TH)$^{+}$ neurons constituted more than 40% of the βIII-tubulin^{+} neurons (Figure 1C). The proportions of βIII-tubulin^{+} TH^{+} neurons to the total population did not change significantly between PARK2 patient lines and control lines, indicating the consistency of DA neuron generation in our protocol. We further characterized TH^{+} neurons with midbrain markers, including forkhead box protein A2 (FOXA2), engrailed-1 (EN1), nur-related factor 1 (NURR1), and G-protein-activated inward rectifier potassium channel 2 (GIRK2) (Hartfield et al., 2014; Xi et al., 2012), further confirming the production of dopamine on day 14 by immunocytochemical analysis (Figure 1D). Because we detected electrophysiological activity in the neurons on day 14 by recording using an Axion MEA system (Figures S2A–S2E), we extended our analysis to demonstrate the functional properties of the neurons by measuring dopamine release. To demonstrate the functional properties of these DA neurons, we measured dopamine release by ELISA. We found that the amount of spontaneous dopamine release was increased during neuronal differentiation (Figure 1E).

(C) Quantitative analyses of cells positive for βIII-tubulin, MAP2, TH, and GFAP. Data represent the means ± SEM (n = 4–6 independent biological replicates).
(D) Characterization of DA neurons from Control A, Control B, *PARK2*$^{-/-}$, PA, and PB at differentiation day 14. Immunocytochemical staining was performed with antibodies against neuronal markers (βIII-tubulin and MAP2), an astrocytic marker (GFAP), a dopaminergic neuron marker (TH), midbrain markers (FOXA2, EN1, NURR1, and GIRK2) and dopamine. Insets are the images of GFAP^{+} signals. Scale bar, 100 μm.
(E) Quantification of dopamine released from control iPSC (Control B)-derived DA neurons. Data represent the means ± SEM (n = 4 independent biological replicates).
(F) A comparison of the levels of dopamine released by DA neurons from Control A and PB on day 14. Data represent the means ± SEM (n = 5 independent biological replicates).
(G) Neurite length was examined in the DA neurons derived from Control A and PARK2 patient-specific NPCs (PB and PA) on day 14. Quantification of the neurite length is shown (right). Data represent the means ± SEM (n = 4–10 independent biological replicates). ***$p < 0.001$ by an unpaired t test. Scale bar, 100 μm.
(H) Neurite length was examined in DA neurons derived from a *PARK2*$^{-/-}$ line and the parental line (Control B) on day 14. Quantification is shown (right). Data represent the means ± SEM (n = 4 independent biological replicates). ***$p < 0.001$ by an unpaired t test. Scale bar, 100 μm.
(I) Elevated intracellular oxidative stress in PARK2- and *PARK2*$^{-/-}$-DA neurons. Intracellular oxidative stress was assessed in DA neurons on day 14 with an indicator, CellROX. Representative images of CellROX^{+} signals in the DA neurons are shown (left). Insets are the images of CellROX^{+} signals. Scale bar, 20 μm. Quantification is shown (right). Data represent the means ± SEM (n = 3 independent biological replicates). *$p < 0.05$, **$p < 0.01$, ***$p < 0.001$ by Dunnett's multiple comparison test.
(J) The abundance of apoptotic cells was increased in PARK2- and *PARK2*$^{-/-}$-DA neurons on day 14. Representative images of cleaved caspase-3 (CASP3) immunostaining are shown (left). Scale bar, 20 μm. Quantification is shown (right). Data represent the means ± SEM (n = 7–10 independent biological replicates). **$p < 0.01$, ***$p < 0.001$ by Dunnett's multiple comparison test.

Furthermore, no difference was observed between the DA neurons derived from a control line (Cont A) and a PARK2 line (PB) in regard to dopamine release on day 14 (Figure 1F). Taken together, we could develop an efficient and robust differentiation protocol for the generation of midbrain DA neurons from NPCs.

Moreover, given the observed axonal degeneration of DA neurons of the SN in PD patients (Cheng et al., 2010) and reduced morphological complexity of iPSC-derived DA neurons from PD patients (Ren et al., 2015), we examined the neuronal morphology by measuring the neurite length of DA neurons on day 14 (Figure S2F). As shown in Figure 1G, the neurons derived from PARK2 iPSC-NPCs exhibited reduced neuronal processes compared with those of a control iPSC-NPC line as judged by βIII-tubulin staining. This phenotype was also observed in the DA neurons derived from the *PARK2*$^{-/-}$ iPSC line (Figure 1H), indicating that the morphological abnormality of the DA neurons was caused by a mutation in the *PARK2* gene. Oxidative stress plays a key role in the selective degeneration of SN DA neurons in PD (Dias et al., 2013; Du et al., 2018), and we previously showed the presence of elevated oxidative stress levels in PARK2 iPSC-derived neurons (Imaizumi et al., 2012). Therefore, we next investigated whether the oxidative stress levels were also increased in the PARK2- or *PARK2*$^{-/-}$-DA neurons by using CellROX Green Reagent to measure the intracellular oxidative stress levels (Figure S2F). The proportion of CellROX^{+} cells was significantly increased in the PARK2- and *PARK2*$^{-/-}$-DA neurons compared with the control DA neurons, indicating increased intracellular oxidative stress levels in the DA neurons in PD (Figures 1I and S2G), consistent with a previous report (Chung et al., 2016). We further investigated the cell viability of PARK2- or *PARK2*$^{-/-}$-DA neurons by the expression of cleaved caspase-3 (CASP3), which labels apoptotic cells (Figure S2F). As shown in Figures 1J, S2H, and S3B, we found a significant increase in the apoptotic cell population among PARK2- and *PARK2*$^{-/-}$-DA neurons. Although we demonstrated increased oxidative stress and apoptosis in the PARK2-DA neurons after the purification of DA neuronal progenitors using fluorescence-activated cell sorting (Suzuki et al., 2017), our current procedures enabled us to simplify the differentiation steps to generate DA neurons. Utilizing isogenic *PARK2*$^{-/-}$ lines in parallel with patient-derived lines, we could demonstrate that loss of *Parkin* function in PD triggered cellular stress and cell death in DA neurons.

PARK2-Dopaminergic Neurons Showed Increased Susceptibility to Rotenone-Induced Stress

Because it has been reported that mitochondrial respiratory chain complex I activity is reduced in the brains of PD patients (Schapira et al., 1989; Winklhofer and Haass, 2010), we used rotenone, a mitochondrial complex I inhibitor (Du et al., 2016), to mimic environmental toxicity to mitochondria. We speculated that a combination of genetic mutations and environmental toxicity would be important for developing a disease model to suppress the pathological progression of PD. Accordingly, we compared the susceptibility of the control and PARK2-DA neurons to the rotenone treatment. The DA neurons were treated with rotenone (10 μM) for 3 hr or 24 hr and analyzed for intracellular oxidative stress and apoptotic cells, respectively. As expected, the rotenone-exposed PARK2-DA neurons exhibited higher oxidative stress levels than the control DA neurons (Figure 2A). Furthermore, we observed higher rates of CASP3^{+} apoptotic DA neurons in the rotenone-exposed PARK2 lines than in the control lines (Figure 2B). The enhanced apoptosis in the PARK2-DA neurons was further evaluated and confirmed by an *in situ* TUNEL analysis (Figures S3A and S3B). We also detected the vulnerability of the mitochondrial membrane potential among the rotenone-exposed PARK2-DA neurons (Figure S3C). In addition, to determine whether the effect of rotenone was specific to DA neurons, we also performed the same analysis on TH^{-} βIII-tubulin^{+} neurons. Interestingly, TH^{-} neurons were less susceptible to rotenone-induced apoptosis, and there was no significant difference between the control and patient-derived neurons, indicating selective susceptibility of the DA neurons to rotenone-induced stress, which further supports subtype-specific neuronal loss in PD. In the following analysis, given there is an increased dynamic range of disease-related phenotypes by rotenone treatment, we applied the experimental paradigm to proceed with chemical screening to identify the compounds that reduce the disease-related phenotypes.

A Calcium Channel Antagonist Prevented Rotenone-Induced Apoptosis and Rescued Impaired Neurite Outgrowth in PARK2-Dopaminergic Neurons

To evaluate whether *in vitro* disease modeling using PARK2-DA neurons could be used as a system for drug discovery, we further explored the compounds that reduce the vulnerability of DA neurons. To repurpose existing drugs, we conducted an initial screening with an FDA-approved drug library containing 1,165 compounds (Figure 3A) (Shimizu et al., 2017). In the initial screening, 88 compounds were selected because the CASP3 levels in the treated DA neurons were less than 50% of the levels in the neurons treated with only rotenone (Figure 3B). Then, we classified the compounds into several subgroups according to their major pharmacological effects, such as calcium channel antagonists (CCAs), cardiovascular drugs, and antibiotic/antiparasitic drugs (Figure S4A). The category of CCAs was attractive considering the number of confirmed hit compounds. For example, benidipine (a dihydropyridine [DHP]-derived triple L-, N-, and T-type CCA) strongly

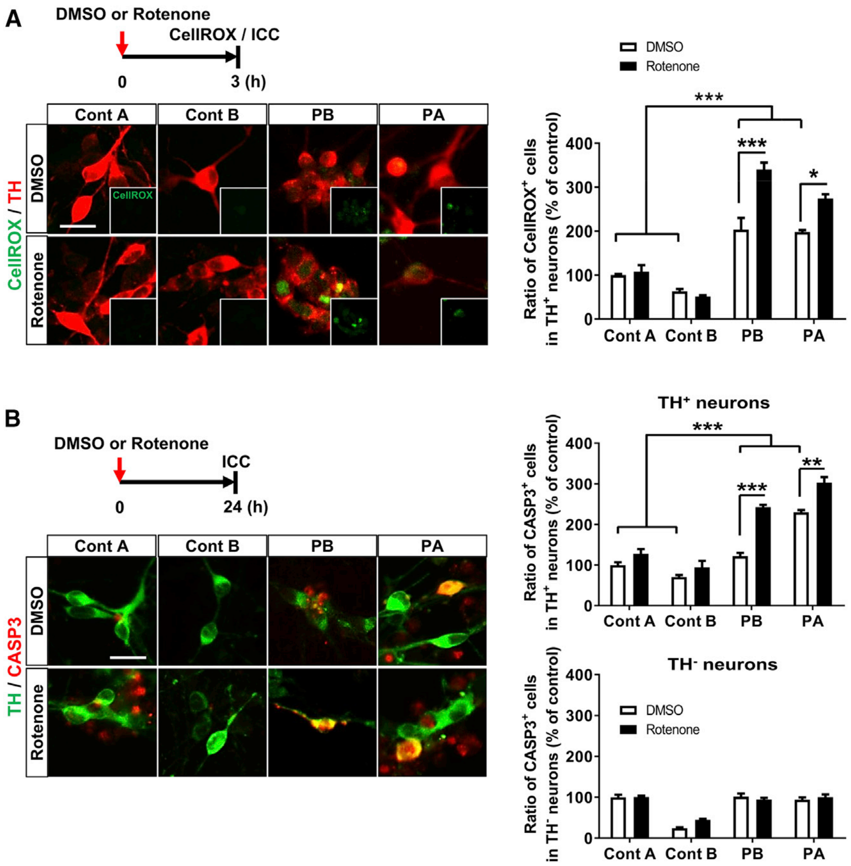

Figure 2. PARK2-Dopaminergic Neurons Showed Increased Susceptibility to Rotenone-Induced Stress

(A) Enhancement of the CellROX$^+$ fraction by rotenone treatment in PARK2-DA neurons on day 14. Representative staining images of CellROX$^+$ cells with or without rotenone exposure (10 μM, 3 hr) are shown (left). Insets are the images of CellROX$^+$ signals. Quantification is shown (right). Data represent the means ± SEM (n = 3–10 independent biological replicates). *$p < 0.05$, ***$p < 0.001$ by Tukey's multiple comparison test. Scale bar, 20 μm.

(B) Immunocytochemical analysis of CASP3$^+$ cells in DA neurons on day 14 with or without rotenone treatment (10 μM, 24 hr). Cells marked as TH$^-$ neurons represent the population of TH$^-$ and βIII-tubulin$^+$ neurons. Quantification of apoptotic cells in TH$^+$ neurons and TH$^-$ neurons. Data represent the means ± SEM (n = 3–6 independent biological replicates). **$p < 0.01$, ***$p < 0.001$ by Tukey's multiple comparison test. Scale bar, 20 μm.

reduced rotenone-induced apoptosis (Figure 3C) (Kopecky et al., 2014; Yao et al., 2006). In addition, cinnarizine (a phenylpiperazine-derived dual L- and T-type CCA) (Figure S4B) (Cohen et al., 1992) and amiodarone (a benzofuran-derived dual L- and T-type CCA) (Cohen et al., 1992) also displayed neuroprotective effects in the PARK2-DA neurons (Figure S4C). Because these compounds exhibited diverse chemical structures while being categorized in the same category, their neuroprotective effects seemed to be mechanism dependent. However, nifedipine (a DHP-derived selective L-type CCA) (Curtis and Scholfield, 2001) and isradipine (a DHP-derived selective L-type CCA) (Ritz et al., 2010), the same DHP analogs as benidipine, failed to prevent rotenone-induced apoptosis (Figures S4D and S4E), indicating that the calcium channel subtype is important for neuroprotective effects against PARK2-DA neurons. This finding is slightly contradictory to the previous observation that L-type calcium channels, especially the CaV1.3 subtype, generate an activity-related oscillatory calcium burden in SN DA neurons, contributing to their degeneration and the pathology of PD (Hurley et al., 2013; Surmeier et al., 2005). Because the presence of T-type calcium channels in DA neurons remains unclear, we conducted the analysis using a selective T-type calcium channel antagonist, ML218 (Xiang et al., 2011). As expected, ML218 suppressed rotenone-induced apoptosis in the PARK2-DA neurons (Figure 3D). These results indicated that T-type calcium channels contribute to rotenone-induced apoptosis in the PARK2-DA neurons. Next, to confirm whether benidipine is neuroprotective, we investigated its effect on neurite abnormalities in PARK2-DA neurons and found that benidipine also rescued impaired neurite outgrowth in PARK2-DA neurons (Figure 3E). Taken together, the results from our screening system using PARK2-DA neurons identified some existing drugs that have potential neuroprotective effects as well as new candidate therapeutic target pathways.

A Calcium Channel Antagonist Displayed Neuroprotective Effects against Dopaminergic Neurons Derived from a PARK6 Patient-Specific iPSC Line

To further validate the effect of benidipine on PD patient-derived DA neurons, we examined its effect on the DA neurons generated from different PD patient-derived DA neurons. We utilized a different form of familial PD with a mutation of PARK6. PARK6 encodes PINK1, a mitochondria-targeted kinase thought to play an important role in mitochondrial homeostasis together with PARKIN (Seibler et al., 2011). Importantly, the DA neurons derived from

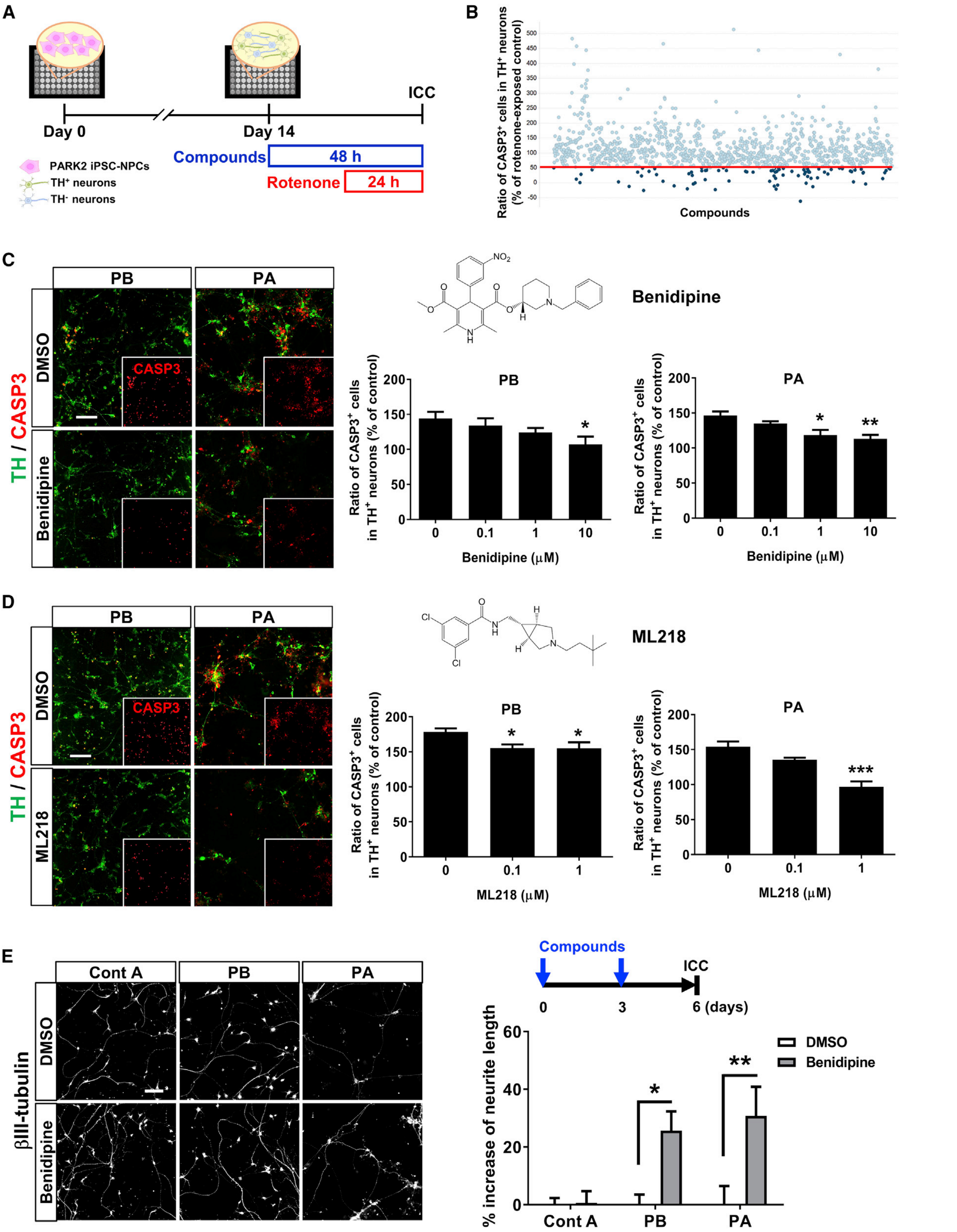

(legend on next page)

PARK6 patient-specific iPSCs were reported to display several PD phenotypes, including cell-type-specific vulnerability and mitochondrial dysfunction (Chung et al., 2016). We generated NPCs from PARK6 patient-specific iPSCs (Shiba-Fukushima et al., 2017) and differentiated them into DA neurons. The PARK6 iPSC-NPCs were also competent with differentiation toward DA neurons, and the PARK6 iPSC-NPC-derived DA neurons (PARK6-DA neurons) displayed several markers of DA neurons (Figure 4A). As observed in the PARK2-DA neurons, the PARK6-DA neurons displayed shortened neurite lengths and elevated oxidative stress levels compared with the control DA neurons (Figures 4B and 4C). Furthermore, the rotenone treatment also enhanced the oxidative stress levels and apoptosis in the PARK6-DA neurons (Figures 4C and 4D), indicating that disease-specific phenotypes could also be reconstructed in DA neurons derived from PARK6 iPSC lines. Finally, we examined the neuroprotective effects of benidipine on PARK6-DA neurons; we found that benidipine protected the DA neurons from rotenone-induced stress in a concentration-dependent manner (Figure 4E) and rescued impaired neurite outgrowth (Figure 4F). Taken together, our results show that the neuroprotective effect of benidipine on PD-derived DA neurons was highly specific to disease-relevant abnormality in PD patients.

Dysregulation of Calcium Homeostasis in PARK2-Dopaminergic Neurons Was Suppressed by Selective T-type Calcium Channel Inhibition

Considering the function of benidipine, it is likely that the dysregulation of cellular calcium homeostasis might result in the vulnerability of DA neurons in PD. Previous works in mouse SN DA neurons have shown that T-type calcium channels are present and functionally important (Dufour et al., 2014; Simms and Zamponi, 2014). Therefore, we examined the expression of the voltage-gated calcium channel in DA neurons. Interestingly, we observed higher expression of the T-type calcium channels (CaV3.1, CaV3.2, and CaV3.3) in PARK2-DA neurons than in control DA neurons (Figures 5A and S5A). We then utilized the calcium indicator dye Fluo-8 AM to examine the intracellular calcium levels in PARK2-derived neurons. As shown in Figure 5B, the neurons derived from the PARK2 iPSC lines had increased resting calcium levels compared with the control line (PARK2 including PB and PA, 174% increase compared with the control; $^{*}p < 0.05$ by an unpaired t test, n = 4–8 independent biological replicates). In addition, we observed significantly increased intracellular calcium levels in the rotenone-treated PARK2 iPSC lines (Figure 5B). Furthermore, benidipine and ML218 inhibited rotenone-induced intracellular calcium increases in the PARK2-derived neurons (Figure 5C), indicating that T-type calcium channels contribute to the dysregulation of calcium homeostasis in PARK2-DA neurons. To gain insight into the functional role of T-type calcium channels, we performed a loss-of-function analysis using small interfering RNAs (siRNAs) against T-type calcium channels and examined their effects on DA neurons. Initially, we examined the expression level of mRNA and protein of each channel after the introduction of siRNAs into the DA neurons and found reduced expression of each subunit of the channel in the DA neurons (Figures 5D and S5C). Then, we introduced the siRNA into the PARK2-DA neurons, exposed them to rotenone, and assessed their intracellular calcium levels using Fluo-8 AM. We found that knockdown of any individual T-type calcium channel subtype resulted in a tendency to decrease the calcium levels (Figure S5E). In addition, immunocytochemical analysis for CASP3 revealed that knockdown of T-type calcium channels suppressed rotenone-induced apoptosis in the PARK2-DA neurons (Figure 5E). These phenotypes were also observed in the DA neurons derived from the *PARK2*$^{-/-}$ iPSC line (Figures S5B, S5D, and 5F). To further validate our findings about the dysregulated expression of T-type calcium channels, we overexpressed T-type calcium channels (CaV3.1 and CaV3.3) into control DA neurons to examine whether a dysregulated intracellular calcium

Figure 3. A Calcium Channel Antagonist Protected against Rotenone-Induced Apoptosis and Rescued Impaired Neurite Outgrowth in PARK2-Dopaminergic Neurons

(A) Experimental paradigm to identify chemical compounds targeting PD. PARK2-DA neurons (PB) were exposed to rotenone (10 μM) or DMSO for 24 hr. In the compound screening, the DA neurons were treated with test compounds (10 μM) in duplicate for 24 hr prior to rotenone treatment.

(B) Representative data from initial screening. The vertical axis shows the inhibitory effect of the test compounds on rotenone-induced apoptosis. Each dot represents an individual compound. The red line indicates 50% of the control value. Eighty-eight of 1,165 compounds were found to reduce CASP3 levels to <50% (% of rotenone-exposed PARK2-DA neuron count).

(C and D) Protective effects of (C) benidipine and (D) ML218 on rotenone-mediated (10 μM, 24 hr) apoptosis. Representative images of CASP3^{+} cells in DA neurons are shown (left). Insets are the images of CASP3^{+} signals. Data represent the means ± SEM (n = 3–16 independent biological replicates). $^{*}p < 0.05$, $^{**}p < 0.01$, $^{***}p < 0.001$ by Dunnett's multiple comparison test. Scale bar, 100 μm.

(E) Neurite length was examined in the control and PARK2-derived neurons treated with DMSO or benidipine (10 μM) for 6 days. Quantification of the neurite length is shown (right). Data represent the means ± SEM (n = 4 independent biological replicates). $^{*}p < 0.05$, $^{**}p < 0.01$ by a t test with Sidak's correction.

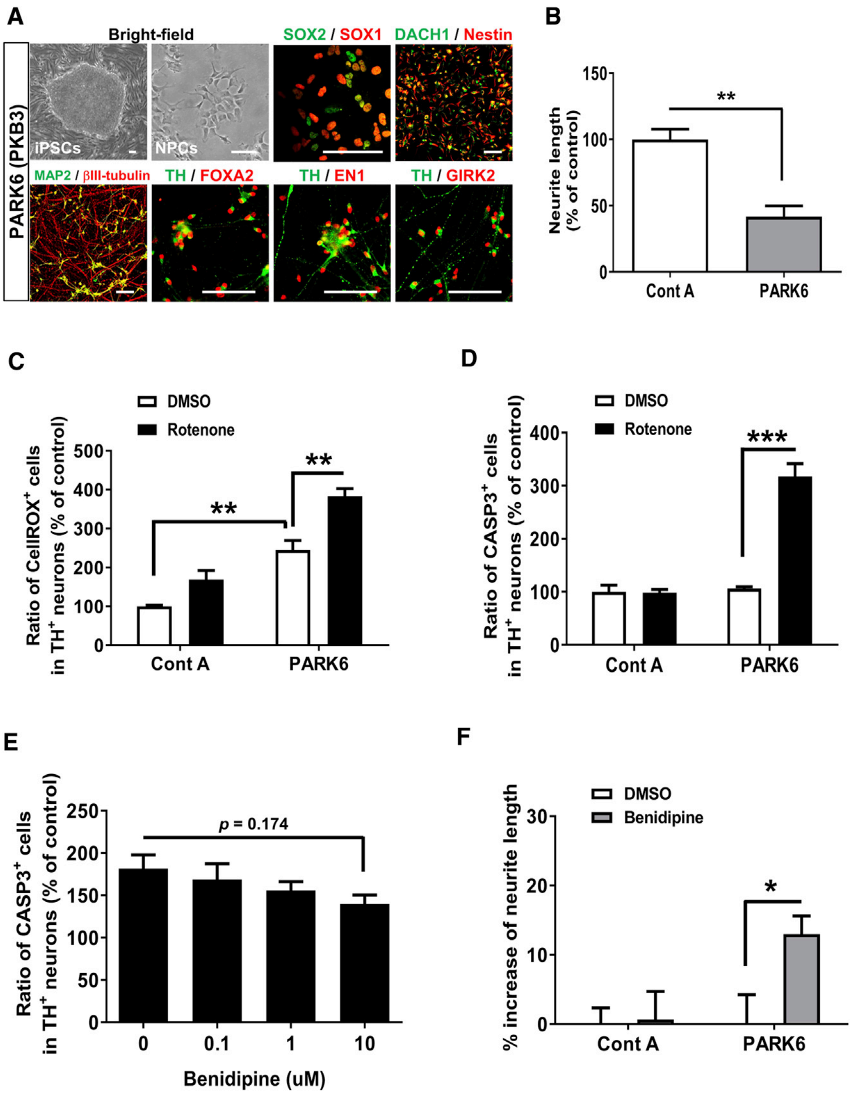

Figure 4. A Calcium Channel Antagonist Displayed Neuroprotective Effects in Dopaminergic Neurons Derived from a PARK6 Patient-Specific iPSC Line

(A) Representative images of iPSCs, NPCs, and DA neurons derived from a PARK6 patient with *PINK1* mutations (PKB3). The expression of four neural stem cell-related markers, including SOX1, SOX2, DACH1, and nestin, indicated that the cells are neural stem cells. PARK6-DA neurons on day 14 expressed DA neuron markers, such as βIII-tubulin, MAP2, TH, FOXA2, EN1, and GIRK2. Scale bar, 100 μm.

(B) Reduced neurite length in PARK6-DA neurons on day 14 compared with the control (Control A). Quantification of neurite length is shown. Data represent the means ± SEM ($n = 4$ independent biological replicates). $^{**}p < 0.01$ by an unpaired t test.

(C) Immunocytochemical analysis of CellROX$^+$ cells in DA neurons (Control A and PARK6) on day 14 with or without rotenone treatment (10 μM, 3 hr). Data represent the means ± SEM ($n = 3$ independent biological replicates). $^{**}p < 0.01$ by Tukey's multiple comparison test.

(D) Immunocytochemical analysis of CASP3$^+$ cells in DA neurons (Control A and PARK6) on day 14 with or without rotenone treatment (10 μM, 24 hr). Data represent the means ± SEM ($n = 3$ independent biological replicates). $^{***}p < 0.001$ by Tukey's multiple comparison test.

(E) The PARK6-DA neurons were treated with benidipine 24 hr prior to rotenone treatment (10 μM, 24 hr). Data represent the means ± SEM ($n = 6–12$ independent biological replicates). p value was calculated by Dunnett's multiple comparison test.

(F) Neurite length was examined in the control (Control A) and PARK6-DA neurons treated with DMSO or benidipine (10 μM) for 6 days. Data represent the means ± SEM ($n = 4$ independent biological replicates). $^{*}p < 0.05$ by a t test with Sidak's correction.

concentration triggers PD-like phenotypes in the control DA neurons. Interestingly, the overexpression of T-type calcium channels triggered increased susceptibility of the DA neurons to rotenone-induced stress (Figures S5F–S5H). These results suggest that the selective vulnerability of DA neurons to rotenone-induced stress in PARK2 was attributable to the dysregulation of intracellular calcium homeostasis via the T-type calcium channels.

DISCUSSION

Because various therapeutic strategies have failed to yield a promising treatment for the disease processes of PD (Athauda and Foltynie, 2015), it is important to establish model systems for developing more effective therapeutic interventions; such systems would be a bridge between animal models and human patients. A postmortem analysis is informative regarding the end-stage pathology of PD, but understanding the early molecular changes associated with the initiation of PD is required to develop improved therapies to halt the progression of the disease. A model system that faithfully reflects the pathogenesis of PD would support the development of more effective therapies. In this respect, PD patient-derived iPSCs represent a useful tool for *in vitro* disease modeling. In parallel with the analysis using isogenic *PARK2*$^{-/-}$ iPSCs (Figures 1 and S1), we established a robust and well-controlled *in vitro* culture

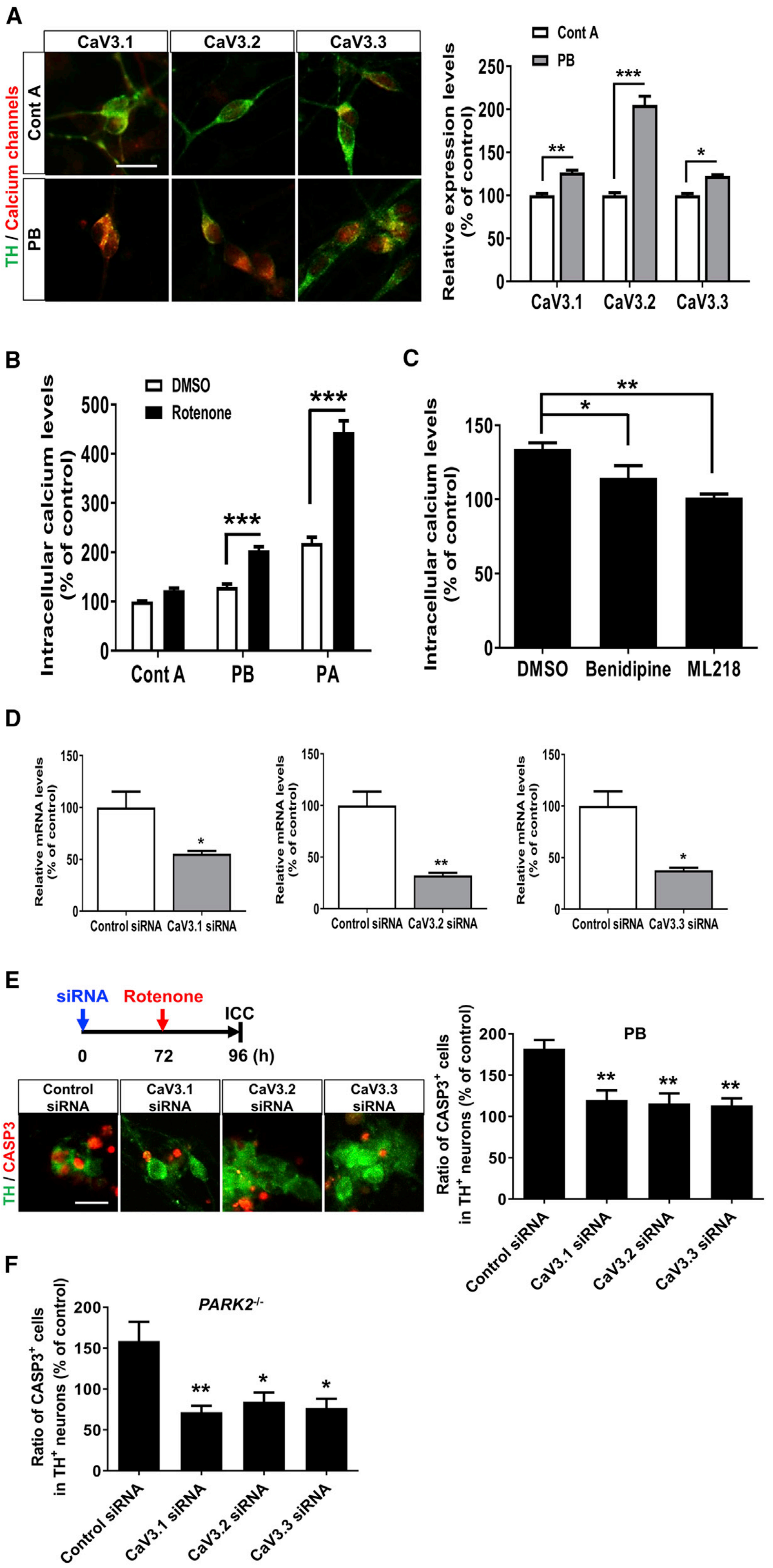

Figure 5. Dysregulation of Calcium Homeostasis in PARK2-Dopaminergic Neurons was Suppressed by Selective T-type Calcium Channel Inhibition

(A) Immunocytochemistry was performed on day 14 for the T-type calcium channel subtypes (CaV3.1, CaV3.2, and CaV3.3). Representative image of calcium channel subtypes in DA neurons (left). The relative expression levels of calcium channel subtypes are shown (right). Data represent the means ± SEM (n = 4 independent biological replicates). $^{*}p < 0.05$, $^{**}p < 0.01$, $^{***}p < 0.001$ by a t test with Sidak's correction. Scale bar, 20 μm.

(B) Measurement of intracellular calcium levels of neurons (Control A, PB, and PA) on day 14 with or without rotenone treatment (10 μM, 24 hr) by the fluorescent calcium indicator Fluo-8 AM. Data represent the means ± SEM (n = 3–4 independent biological replicates). $^{***}p < 0.001$ by Tukey's multiple comparison test.

(C) The effect of CCAs on the intracellular calcium concentration in PARK2-DA neurons (PB). The cells were exposed to benidipine (1 μM) or ML218 (1 μM) 24 hr prior to rotenone treatment. Data represent the means ± SEM (n = 3–6 independent biological replicates). $^{*}p < 0.05$, $^{**}p < 0.01$ by Dunnett's multiple comparison test.

(D) Knockdown was performed on day 14 with transient siRNA transfection, Accell siRNA (1 μM) against T-type calcium channel subtypes for 72 hr in the control DA neurons (Control B), and verified with qRT-PCR. Values were normalized to the expression of GAPDH, and changes in mRNA levels were measured relative to non-targeting control siRNA levels. Data represent the means ± SEM (n = 3 independent biological replicates). $^{*}p < 0.05$, $^{**}p < 0.01$ by an unpaired t test.

(E and F) Requirement of T-type calcium channel subtypes for the vulnerability of (E) PARK2 (PB)- and (F) *PARK2*$^{-/-}$-DA neurons. The cells were transfected with siRNA against the various T-type calcium channel subtypes for 72 hr prior to rotenone treatment. Immunocytochemical analysis of the CASP3^{+} cells in DA neurons on day 14 with rotenone treatment (10 μM, 24 hr). Representative image of CASP3^{+} cells in PARK2-DA neurons are shown (left). Data represent the means ± SEM (n = 3 independent biological replicates). $^{*}p < 0.05$, $^{**}p < 0.01$ by Dunnett's multiple comparison test. Scale bar, 20 μm.

system and demonstrated several PD-related phenotypes, including neurite abnormalities, elevated oxidative stress, and apoptosis in PARK2- and *PARK2*$^{-/-}$-DA neurons (Figures 1, S2, and S3B). Although several PD-related phenotypes have been reported in PARK2 iPSCs (Imaizumi et al., 2012; Ren et al., 2015; Shaltouki et al., 2015), the frequency of DA neurons within the entire neuronal population in these studies is either unmentioned or less than 15%. In contrast, we have successfully generated DA neurons from iPSC lines, and the DA neurons further exhibited midbrain regional specificity and dopamine production (Figure 1). Recently, we also reported a method for preparing highly enriched DA neurons using fluorescence-activated cell sorting (Suzuki et al., 2017), but the current procedure is more advanced in terms of simplicity and robustness for neuronal differentiation, enabling us to perform screening without complicated work.

Notably, we further extended our study to screen CCAs based on their effects on rotenone-treated PARK2-DA neurons (Figures 3 and S4). It is noteworthy that selectivity for calcium channel subtypes may be associated with differences in neuroprotective effects in different DHP analogs, such as benidipine, nifedipine, and isradipine. We also validated the results obtained from PARK2-DA neurons in the DA neurons derived from PARK6 iPSCs (Figure 4). Mechanistically, loss-of-function mutations in PARK2 or PARK6 have been associated with mitochondrial dysfunction, including reduced ATP production, impaired mitochondrial autophagy, mitophagy, and disruption of intracellular calcium homeostasis (Chung et al., 2016; Scarffe et al., 2014). Mitochondria are important temporal and spatial regulators of intracellular calcium concentrations in neurons, and mitochondrial dysfunction triggers mitochondrial permeability transition pore opening via high mitochondrial calcium levels and mitochondrial depolarization. The opening of pores releases apoptotic factors and leads to apoptosis (Cali et al., 2012). In the present report, we observed selective upregulation of T-type calcium channels in PARK2-DA neurons. Regarding the relationship between calcium channels and PD pathogenesis, SN DA neurons are reported to be autonomous pacemakers that fire action potentials in the absence of excitatory synaptic input; this pacemaker activity is mainly regulated by the L-type calcium channel CaV1.3, as revealed by an animal model (Surmeier et al., 2005). This function is consistent with the potential beneficial effect of certain CCAs—specifically, inhibitors of L-type calcium channels—against PD, as revealed by etiological observations in patients using CCAs as an antihypertensive intervention (Ritz et al., 2010). Although the potential neuroprotective role of isradipine has been reported (Surmeier et al., 2010, 2017), the plasma concentration of isradipine approved for therapy has been reported to be insufficient for the treatment of a PD animal model (Ortner et al., 2017). Furthermore, only modest advantages were achieved in a recent clinical trial (STEADY-PD) (Parkinson Study, 2013). Therefore, the efficacy of isradipine remains controversial, and the results of an ongoing phase 3 clinical trial (STEADY-PD III) are awaited to reach a definitive conclusion about its effectiveness against PD (Surmeier et al., 2017). On the other hand, at least in our experimental model, the dysregulation of L-type calcium channels is less likely to contribute to the pathogenesis of PD. Furthermore, Evans et al. (2017) showed, using mouse brain slices, that SN DA neurons negative for the calcium buffering protein calbindin are highly vulnerable compared with calbindin^{+} SN neurons. In addition, calbindin^{-} DA neurons have a higher density of T-type calcium channels than calbindin^{+} DA neurons. However, because of the limited availability of suitable models, there is no direct evidence linking selective vulnerability with T-type calcium channels in the PD brain to date. Therefore, iPSC-based disease modeling provides a versatile *in vitro* system to evaluate the pathogenesis of neurodegenerative disease. One might wonder about the validity of the concentration of the compound we applied in our experiment compared with the clinical dosage of the compound. As the neuroprotective dose of benidipine (50% inhibitory concentration [IC_{50}] = 1.1 μM; 95% confidence interval, 0.2 to 6.7 μM; n = 9 independent biological replicates) in the rotenone-exposed cell-based assay was found to be similar to the therapeutic range of this drug in clinical use (3.7–7.4 μM as an antihypertensive) (Kopecky et al., 2014), this compound may be suitable for new therapeutic purposes, such as PD treatment, and T-type CCAs are strong candidates for disease-modifying drugs that can alleviate the progressive pathology of PD (Figure 6). Although we utilized DA neurons from PAKR2 and PARK6 iPSCs, we cannot rule out the possibility that the neuroprotective effect of benidipine is specific to DA neurons with an abnormal PINK1-PARKIN pathway. Furthermore, in our experimental model for drug screening, we utilized rotenone to enhance the disease-related phenotypes. Enhancement of the *in vitro* phenotype was also achieved in other chemical compounds such as carbonyl cyanide m-chlorophenylhydrazone (CCCP), an uncoupler that is known to dissipate mitochondrial membrane potential and induced entry of extracellular calcium through L/N-type calcium channels (Gomez-Sanchez et al., 2014). Similar to the effects of rotenone, we observed an increased proportion of TH^{+} neurons co-expressing CASP3, and CCCP-induced cell death was suppressed by the addition of benidipine (Figure S6). Therefore, future research will clarify the effect of benidipine on other types of PD patient-derived DA neurons.

Because dysregulated mitochondrial function occurs ubiquitously in the PD brain, additional cell-intrinsic

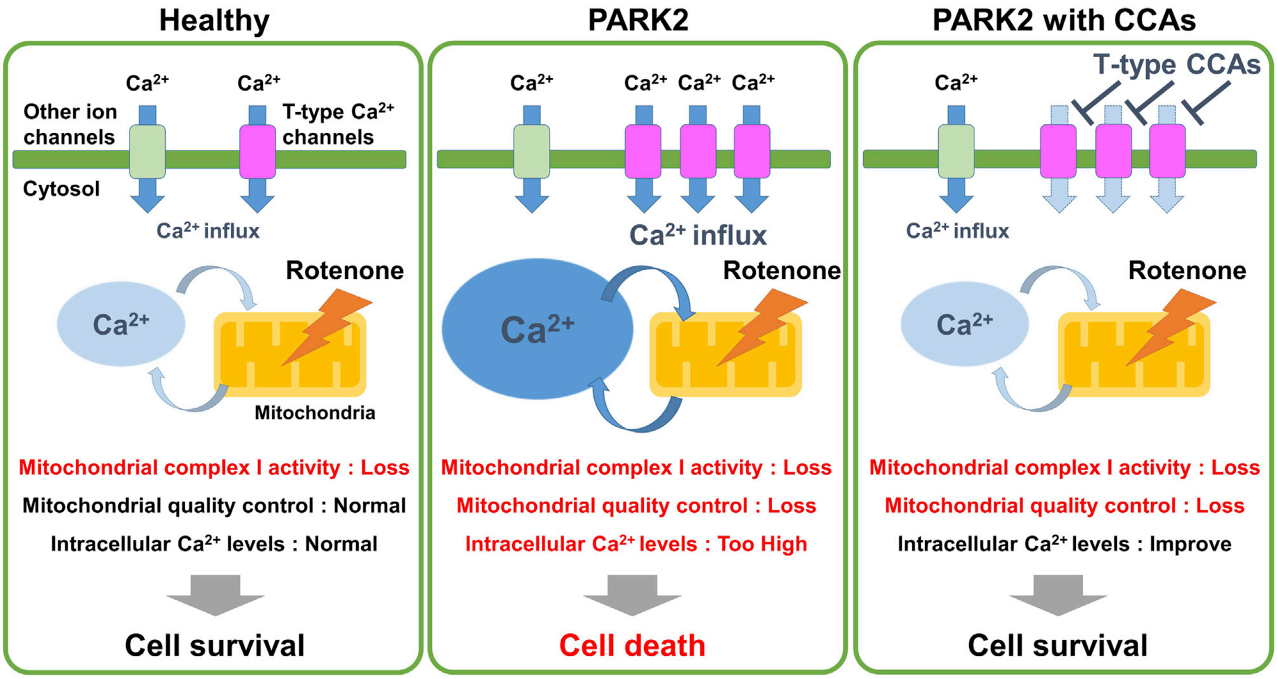

Figure 6. Calcium Homeostasis Dysregulation Triggers Selective Vulnerability of Dopaminergic Neurons to Rotenone-Induced Stress in PARK2

Schematics of calcium homeostasis dysregulation in PARK2-DA neurons. When normal DA neurons were exposed to rotenone, damaged mitochondria were removed by Parkin- and PINK1-mediated mitophagy, allowing normal function to be maintained. However, mitochondrial function could not be restored due to the abnormality of the mitochondrial quality control mechanism in rotenone-exposed PARK2-DA neurons. Therefore, intracellular calcium overload promoted mitochondrial dysfunction and caused cell death specifically in DA neurons. Selective inhibition of T-type calcium channels suppressed the increase in intracellular calcium levels and cell death.

circumstances in midbrain DA neurons might determine cell-type-specific vulnerability, triggering selective death of DA neurons in PD patients. For example, transcriptional regulation of T-type calcium channels in DA neurons is of interest based on our findings that the PARK2-DA neurons expressed more T-type calcium channels (Figures 5 and S5). In contrast with increased intracellular calcium levels after rotenone treatment (Figure 5B), the mRNA level of calcium channels did not change after rotenone treatment, indicating that transcriptional regulation is less likely to contribute to dysregulated intracellular calcium levels in PARK2-DA neurons. Because there is limited information to date on the regulation of those channels, we would like to address this matter experimentally in our future studies. Given that the dysregulation of calcium homeostasis plays a pivotal role in the pathogenesis of several neurodegenerative diseases, including PD (Bezprozvanny, 2010; Surmeier et al., 2010), Alzheimer disease (Bezprozvanny, 2010; Magi et al., 2016), Huntington disease (Bezprozvanny, 2010; Kolobkova et al., 2017), and amyotrophic lateral sclerosis (Bezprozvanny, 2010; Leal and Gomes, 2015), it would be important to unravel the contribution of dysregulated calcium channels in these diseases and how a dysregulated calcium concentration triggers selective neuronal deaths in other types of neuronal subsets.

In summary, our study indicates that PARK2-DA neurons provide an *in vitro* disease model that recapitulates several PD-related disease phenotypes; this model can be used to investigate the pathogenesis of the disease and search for potential therapeutic targets for PD.

EXPERIMENTAL PROCEDURES

A detailed description of the experimental procedures is given in the Supplemental Information.

Generation of Patient-Specific and Isogenic iPSC-Derived Neural Progenitor Cells

The iPSC line 201B7 (Takahashi et al., 2007), PARK2 iPSC lines PA9 and PB2 (Imaizumi et al., 2012), PARK2-deficient iPSC lines (B7PA21) (Suda et al., 2018), and PARK6 iPSC line PKB3 (Shiba-Fukushima et al., 2017) were established and maintained as previously described. The NPCs were established from the 201B7, PA9,

PB2, PKB3, and B7PA21 iPSC lines as previously described (Falk et al., 2012).

Compound Screening

We used an FDA-approved drug library consisting of 1,165 biologically active compounds (Shimizu et al., 2017). The PARK2-DA neurons were incubated for 48 hr in the absence or presence of each compound (10 μM); subsequently, rotenone (10 μM) treatment was conducted for 24 hr (Figure 3A).

Statistical Analysis

Statistical analysis was performed using GraphPad Prism 7.0 (GraphPad Software, La Jolla, CA). The data are described as the means ± standard error of the mean (SEM). Unpaired t tests were used for pairwise comparisons between the groups. One-way analysis of variance (ANOVA) followed by Dunnett's test and two-way ANOVA followed by either Tukey's test or a t test with Sidak's correction were applied for multiple comparisons. The results were considered statistically significant when $p < 0.05$. The IC_{50} was also calculated using GraphPad Prism.

Ethics

All the experimental procedures were approved by the Keio University School of Medicine Ethics committee (Approval Number: 20080016).

SUPPLEMENTAL INFORMATION

Supplemental Information includes Supplemental Experimental Procedures, six figures, and two tables and can be found with this article online at https://doi.org/10.1016/j.stemcr.2018.09.006.

AUTHOR CONTRIBUTIONS

Study concept and design: Y.T., J.K., and H.O.; data acquisition and analysis: Y.T., Y.I., M.S., T.A.-N., S.B., M.C., T.S., K.Y., H.S., and N.H.; drafting the manuscript and figures: Y.T., M.I., K.T., J.K., and H.O.; and reviewing and editing the manuscript: all authors.

ACKNOWLEDGMENTS

We would like to thank Dr. Austin Smith (University of Cambridge) for providing the AF22 cells and Dr. Shinya Yamanaka (Kyoto University) for providing the 201B7. We also thank the members of Okano's laboratory. This work was supported by JSPS KAKENHI grants JP16K15240 and JP26713047 (to J.K), funding from Eisai Co., Ltd. (to H.O.), the Research Project for Practical Application of Regenerative Medicine from the Japan Agency for Medical Research and Development (AMED) (grant no. 15bk0104009h0003 to J.K.), and the Research Center Network for Realization of Regenerative Medicine (Acceleration Program for Intractable Diseases Research Utilizing Disease-specific iPS Cells) from the AMED (grant no. 17bm0804003h0001 to H.O.). H.O. is a compensated scientific consultant for SanBio and K Pharma. Y.T., Y.I., M.S., Y.K., M.I., and K.T. are employed by Eisai. The other authors indicated no potential conflicts of interest.

Received: February 17, 2018
Revised: September 17, 2018
Accepted: September 18, 2018
Published: October 18, 2018

REFERENCES

Athauda, D., and Foltynie, T. (2015). The ongoing pursuit of neuroprotective therapies in Parkinson disease. Nat. Rev. Neurol. *11*, 25–40.

Bezprozvanny, I.B. (2010). Calcium signaling and neurodegeneration. Acta Naturae *2*, 72–82.

Blesa, J., and Przedborski, S. (2014). Parkinson's disease: animal models and dopaminergic cell vulnerability. Front. Neuroanat. *8*, 155.

Cali, T., Ottolini, D., and Brini, M. (2012). Mitochondrial Ca(2+) and neurodegeneration. Cell Calcium *52*, 73–85.

Cheng, H.C., Ulane, C.M., and Burke, R.E. (2010). Clinical progression in Parkinson disease and the neurobiology of axons. Ann. Neurol. *67*, 715–725.

Chung, S.Y., Kishinevsky, S., Mazzulli, J.R., Graziotto, J., Mrejeru, A., Mosharov, E.V., Puspita, L., Valiulahi, P., Sulzer, D., Milner, T.A., et al. (2016). Parkin and PINK1 patient iPSC-derived midbrain dopamine neurons exhibit mitochondrial dysfunction and α-synuclein accumulation. Stem Cell Reports *7*, 664–677.

Cohen, C.J., Spires, S., and Van Skiver, D. (1992). Block of T-type Ca channels in Guinea pig atrial cells by antiarrhythmic agents and Ca channel antagonists. J. Gen. Physiol. *100*, 703–728.

Curtis, T.M., and Scholfield, C.N. (2001). Nifedipine blocks Ca^{2+} store refilling through a pathway not involving L-type Ca^{2+} channels in rabbit arteriolar smooth muscle. J. Physiol. *532*, 609–623.

Damier, P., Hirsch, E.C., Agid, Y., and Graybiel, A.M. (1999). The substantia nigra of the human brain. II. Patterns of loss of dopamine-containing neurons in Parkinson's disease. Brain *122* (Pt 8), 1437–1448.

Dias, V., Junn, E., and Mouradian, M.M. (2013). The role of oxidative stress in Parkinson's disease. J. Parkinsons Dis. *3*, 461–491.

Drouin-Ouellet, J., and Barker, R.A. (2012). Parkinson's disease in a dish: what patient specific-reprogrammed somatic cells can tell us about Parkinson's disease, if anything? Stem Cells Int. *2012*, 926147.

Du, F., Yu, Q., Chen, A., Chen, D., and Yan, S.S. (2018). Astrocytes attenuate mitochondrial dysfunctions in human dopaminergic neurons derived from iPSC. Stem Cell Reports *10*, 366–374.

Du, F., Yu, Q., Shijun, Y., Doris, C., and Yan, S.S. (2016). Development and dynamic regulation of mitochondrial network in human midbrain dopaminergic neurons differentiated from iPSCs. Stem Cell Reports *7*, 678–692.

Du, F., Yu, Q., Yan, S., Hu, G., Lue, L.F., Walker, D.G., Wu, L., Yan, S.F., Tieu, K., and Yan, S.S. (2017). PINK1 signalling rescues amyloid pathology and mitochondrial dysfunction in Alzheimer's disease. Brain *140*, 3233–3251.

Dufour, M.A., Woodhouse, A., and Goaillard, J.M. (2014). Somatodendritic ion channel expression in substantia nigra pars

compacta dopaminergic neurons across postnatal development. J. Neurosci. Res. *92*, 981–999.

Evans, R.C., Zhu, M., and Khaliq, Z.M. (2017). Dopamine inhibition differentially controls excitability of substantia nigra dopamine neuron subpopulations through T-type calcium channels. J. Neurosci. *37*, 3704–3720.

Falk, A., Koch, P., Kesavan, J., Takashima, Y., Ladewig, J., Alexander, M., Wiskow, O., Tailor, J., Trotter, M., Pollard, S., et al. (2012). Capture of neuroepithelial-like stem cells from pluripotent stem cells provides a versatile system for in vitro production of human neurons. PLoS One *7*, e29597.

Funayama, M., Hasegawa, K., Kowa, H., Saito, M., Tsuji, S., and Obata, F. (2002). A new locus for Parkinson's disease (PARK8) maps to chromosome 12p11.2-q13.1. Ann. Neurol. *51*, 296–301.

Gomez-Sanchez, R., Gegg, M.E., Bravo-San Pedro, J.M., Niso-Santano, M., Alvarez-Erviti, L., Pizarro-Estrella, E., Gutierrez-Martin, Y., Alvarez-Barrientos, A., Fuentes, J.M., Gonzalez-Polo, R.A., et al. (2014). Mitochondrial impairment increases FL-PINK1 levels by calcium-dependent gene expression. Neurobiol. Dis. *62*, 426–440.

Hartfield, E.M., Yamasaki-Mann, M., Ribeiro Fernandes, H.J., Vowles, J., James, W.S., Cowley, S.A., and Wade-Martins, R. (2014). Physiological characterisation of human iPS-derived dopaminergic neurons. PLoS One *9*, e87388.

Hurley, M.J., Brandon, B., Gentleman, S.M., and Dexter, D.T. (2013). Parkinson's disease is associated with altered expression of CaV1 channels and calcium-binding proteins. Brain *136*, 2077–2097.

Imaizumi, Y., Okada, Y., Akamatsu, W., Koike, M., Kuzumaki, N., Hayakawa, H., Nihira, T., Kobayashi, T., Ohyama, M., Sato, S., et al. (2012). Mitochondrial dysfunction associated with increased oxidative stress and α-synuclein accumulation in PARK2 iPSC-derived neurons and postmortem brain tissue. Mol. Brain *5*, 35.

Kalia, L.V., and Lang, A.E. (2015). Parkinson's disease. Lancet *386*, 896–912.

Kitada, T., Asakawa, S., Hattori, N., Matsumine, H., Yamamura, Y., Minoshima, S., Yokochi, M., Mizuno, Y., and Shimizu, N. (1998). Mutations in the parkin gene cause autosomal recessive juvenile parkinsonism. Nature *392*, 605–608.

Kolobkova, Y.A., Vigont, V.A., Shalygin, A.V., and Kaznacheyeva, E.V. (2017). Huntington's disease: calcium dyshomeostasis and pathology models. Acta Naturae *9*, 34–46.

Kopecky, B.J., Liang, R., and Bao, J. (2014). T-type calcium channel blockers as neuroprotective agents. Pflugers Arch. *466*, 757–765.

Leal, S.S., and Gomes, C.M. (2015). Calcium dysregulation links ALS defective proteins and motor neuron selective vulnerability. Front. Cell Neurosci. *9*, 225.

Magi, S., Castaldo, P., Macri, M.L., Maiolino, M., Matteucci, A., Bastioli, G., Gratteri, S., Amoroso, S., and Lariccia, V. (2016). Intracellular calcium dysregulation: implications for Alzheimer's disease. Biomed. Res. Int. *2016*, 6701324.

Mizuno, Y., Hattori, N., Kubo, S., Sato, S., Nishioka, K., Hatano, T., Tomiyama, H., Funayama, M., Machida, Y., and Mochizuki, H. (2008). Progress in the pathogenesis and genetics of Parkinson's disease. Philos. Trans. R. Soc. Lond. B Biol. Sci. *363*, 2215.

Narendra, D., Walker, J.E., and Youle, R. (2012). Mitochondrial quality control mediated by PINK1 and Parkin: links to parkinsonism. Cold Spring Harb. Perspect. Biol. *4*. https://doi.org/10.1101/cshperspect.a011338.

Okano, H., and Yamanaka, S. (2014). iPS cell technologies: significance and applications to CNS regeneration and disease. Mol. Brain *7*, 22.

Ortner, N.J., Bock, G., Dougalis, A., Kharitonova, M., Duda, J., Hess, S., Tuluc, P., Pomberger, T., Stefanova, N., Pitterl, F., et al. (2017). Lower affinity of isradipine for L-Type Ca(2+) channels during Substantia Nigra Dopamine neuron-like activity: implications for neuroprotection in Parkinson's disease. J. Neurosci. *37*, 6761–6777.

Parkinson Study, G. (2013). Phase II safety, tolerability, and dose selection study of isradipine as a potential disease-modifying intervention in early Parkinson's disease (STEADY-PD). Mov. Disord. *28*, 1823–1831.

Polymeropoulos, M.H., Lavedan, C., Leroy, E., Ide, S.E., Dehejia, A., Dutra, A., Pike, B., Root, H., Rubenstein, J., Boyer, R., et al. (1997). Mutation in the alpha-synuclein gene identified in families with Parkinson's disease. Science *276*, 2045.

Ren, Y., Jiang, H., Hu, Z., Fan, K., Wang, J., Janoschka, S., Wang, X., Ge, S., and Feng, J. (2015). Parkin mutations reduce the complexity of neuronal processes in iPSC-derived human neurons. Stem Cells *33*, 68–78.

Ritz, B., Rhodes, S.L., Qian, L., Schernhammer, E., Olsen, J., and Friis, S. (2010). L-type calcium channel blockers and Parkinson disease in Denmark. Ann. Neurol. *67*, 600–606.

Scarffe, L.A., Stevens, D.A., Dawson, V.L., and Dawson, T.M. (2014). Parkin and PINK1: much more than mitophagy. Trends Neurosci. *37*, 315–324.

Schapira, A.H., Cooper, J.M., Dexter, D., Jenner, P., Clark, J.B., and Marsden, C.D. (1989). Mitochondrial complex I deficiency in Parkinson's disease. Lancet *1*, 1269.

Seibler, P., Graziotto, J., Jeong, H., Simunovic, F., Klein, C., and Krainc, D. (2011). Mitochondrial Parkin recruitment is impaired in neurons derived from mutant PINK1 induced pluripotent stem cells. J. Neurosci. *31*, 5970–5976.

Shaltouki, A., Sivapatham, R., Pei, Y., Gerencser, A.A., Momčilović, O., Rao, M.S., and Zeng, X. (2015). Mitochondrial alterations by PARKIN in dopaminergic neurons using PARK2 patient-specific and PARK2 knockout isogenic iPSC lines. Stem Cell Reports *4*, 847–859.

Shiba-Fukushima, K., Ishikawa, K.I., Inoshita, T., Izawa, N., Takanashi, M., Sato, S., Onodera, O., Akamatsu, W., Okano, H., Imai, Y., et al. (2017). Evidence that phosphorylated ubiquitin signaling is involved in the etiology of Parkinson's disease. Hum. Mol. Genet. *26*, 3172–3185.

Shimizu, T., Kamel, W.A., Yamaguchi-Iwai, S., Fukuchi, Y., Muto, A., and Saya, H. (2017). Calcitriol exerts an anti-tumor effect in osteosarcoma by inducing the endoplasmic reticulum stress response. Cancer Sci. *108*, 1793–1802.

Simms, B.A., and Zamponi, G.W. (2014). Neuronal voltage-gated calcium channels: structure, function, and dysfunction. Neuron *82*, 24–45.

Suda, Y., Kuzumaki, N., Sone, T., Narita, M., Tanaka, K., Hamada, Y., Iwasawa, C., Shibasaki, M., Maekawa, A., Matsuo, M., et al. (2018). Down-regulation of ghrelin receptors on dopaminergic neurons in the substantia nigra contributes to Parkinson's disease-like motor dysfunction. Mol. Brain *11*, 6.

Surmeier, D.J., Guzman, J.N., and Sanchez-Padilla, J. (2010). Calcium, cellular aging, and selective neuronal vulnerability in Parkinson's disease. Cell Calcium *47*, 175–182.

Surmeier, D.J., Halliday, G.M., and Simuni, T. (2017). Calcium, mitochondrial dysfunction and slowing the progression of Parkinson's disease. Exp. Neurol. *298*, 202–209.

Surmeier, D.J., Mercer, J.N., and Chan, C.S. (2005). Autonomous pacemakers in the basal ganglia: who needs excitatory synapses anyway? Curr. Opin. Neurobiol. *15*, 312–318.

Suzuki, S., Akamatsu, W., Kisa, F., Sone, T., Ishikawa, K.-i., Kuzumaki, N., Katayama, H., Miyawaki, A., Hattori, N., and Okano, H. (2017). Efficient induction of dopaminergic neuron differentiation from induced pluripotent stem cells reveals impaired mitophagy in PARK2 neurons. Biochem. Biophys. Res. Commun. *483*, 88–93.

Takahashi, K., Tanabe, K., Ohnuki, M., Narita, M., Ichisaka, T., Tomoda, K., and Yamanaka, S. (2007). Induction of pluripotent stem cells from adult human fibroblasts by defined factor. Cell *131*, 861–872.

Valente, E.M., Abou-Sleiman, P.M., Caputo, V., Muqit, M.M., Harvey, K., Gispert, S., Ali, Z., Del Turco, D., Bentivoglio, A.R., Healy, D.G., et al. (2004). Hereditary early-onset Parkinson's disease caused by mutations in PINK1. Science *304*, 1158–1160.

Verstraeten, A., Theuns, J., and Van Broeckhoven, C. (2015). Progress in unraveling the genetic etiology of Parkinson disease in a genomic era. Trends Genet. *31*, 140–149.

Winklhofer, K.F., and Haass, C. (2010). Mitochondrial dysfunction in Parkinson's disease. Biochim. Biophys. Acta *1802*, 29–44.

Xi, J., Liu, Y., Liu, H., Chen, H., Emborg, M.E., and Zhang, S.C. (2012). Specification of midbrain dopamine neurons from primate pluripotent stem cells. Stem Cells *30*, 1655–1663.

Xiang, Z., Thompson, A.D., Brogan, J.T., Schulte, M.L., Melancon, B.J., Mi, D., Lewis, L.M., Zou, B., Yang, L., Morrison, R., et al. (2011). The discovery and characterization of ML218: a novel, centrally active T-type calcium channel inhibitor with robust effects in STN neurons and in a rodent model of Parkinson's disease. ACS Chem. Neurosci. *2*, 730–742.

Yao, K., Nagashima, K., and Miki, H. (2006). Pharmacological, pharmacokinetic, and clinical properties of benidipine hydrochloride, a novel, long-acting calcium channel blocker. J. Pharmacol. Sci. *100*, 243–261.

Zimprich, A., Biskup, S., Leitner, P., Lichtner, P., Farrer, M., Lincoln, S., Kachergus, J., Hulihan, M., Uitti, R.J., Calne, D.B., et al. (2004). Mutations in LRRK2 cause autosomal-dominant parkinsonism with pleomorphic pathology. Neuron *44*, 601–607.

Stem Cell Reports, Volume *11*

Supplemental Information

T-type Calcium Channels Determine the Vulnerability of Dopaminergic Neurons to Mitochondrial Stress in Familial Parkinson Disease

Yoshikuni Tabata, Yoichi Imaizumi, Michiko Sugawara, Tomoko Andoh-Noda, Satoe Banno, MuhChyi Chai, Takefumi Sone, Kazuto Yamazaki, Masashi Ito, Kappei Tsukahara, Hideyuki Saya, Nobutaka Hattori, Jun Kohyama, and Hideyuki Okano

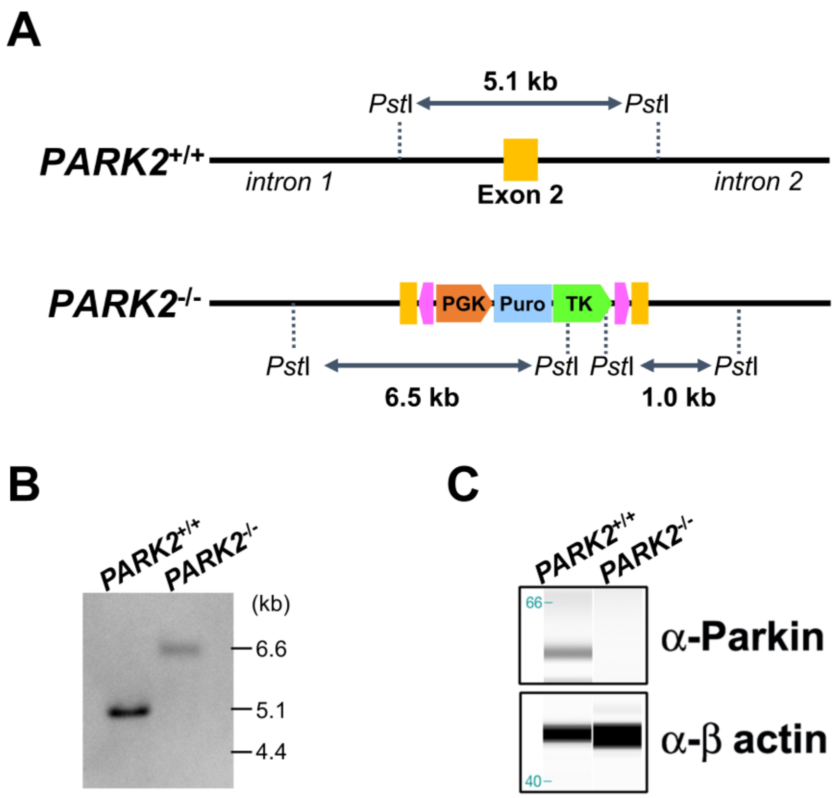
A
5.1 kb
PstI
PstI
PARK2+/+
intron 1
Exon 2
intron 2
PARK2-/-
PGK
Puro
TK
PstI
PstI
PstI
PstI
6.5 kb
1.0 kb
B
PARK2+/+
PARK2-/-
(kb)
6.6
5.1
4.4
C
PARK2+/+
PARK2-/-
66
40
α-Parkin
α-β actin

Figure S1. Generation of Isogenic *PARK2*$^{-/-}$ iPSC-derived Neural Progenitor Cells (Related to Figure 1)

(A) Design of *PARK2* gene knock-out by CRISPR-Cas9 method and construction of plasmid vectors. To generate *PARK2*-deficient iPSC line, we used CRISPR-Cas9 and a targeting donor vector with puromycin resistant gene. After selection with puromycin, isogenic *PARK2*$^{-/-}$ iPSC lines (B7PA21) was identified. (B) To confirm homologous recombination in *PARK2* gene locus, Southern-blotting analysis was used. *PARK2*$^{-/-}$ showed correct 6.5 kb band. (C) The expression of target proteins was determined by the capillary electrophoresis immunoassay using the Wes size-based Simple Western system (ProteinSimple).

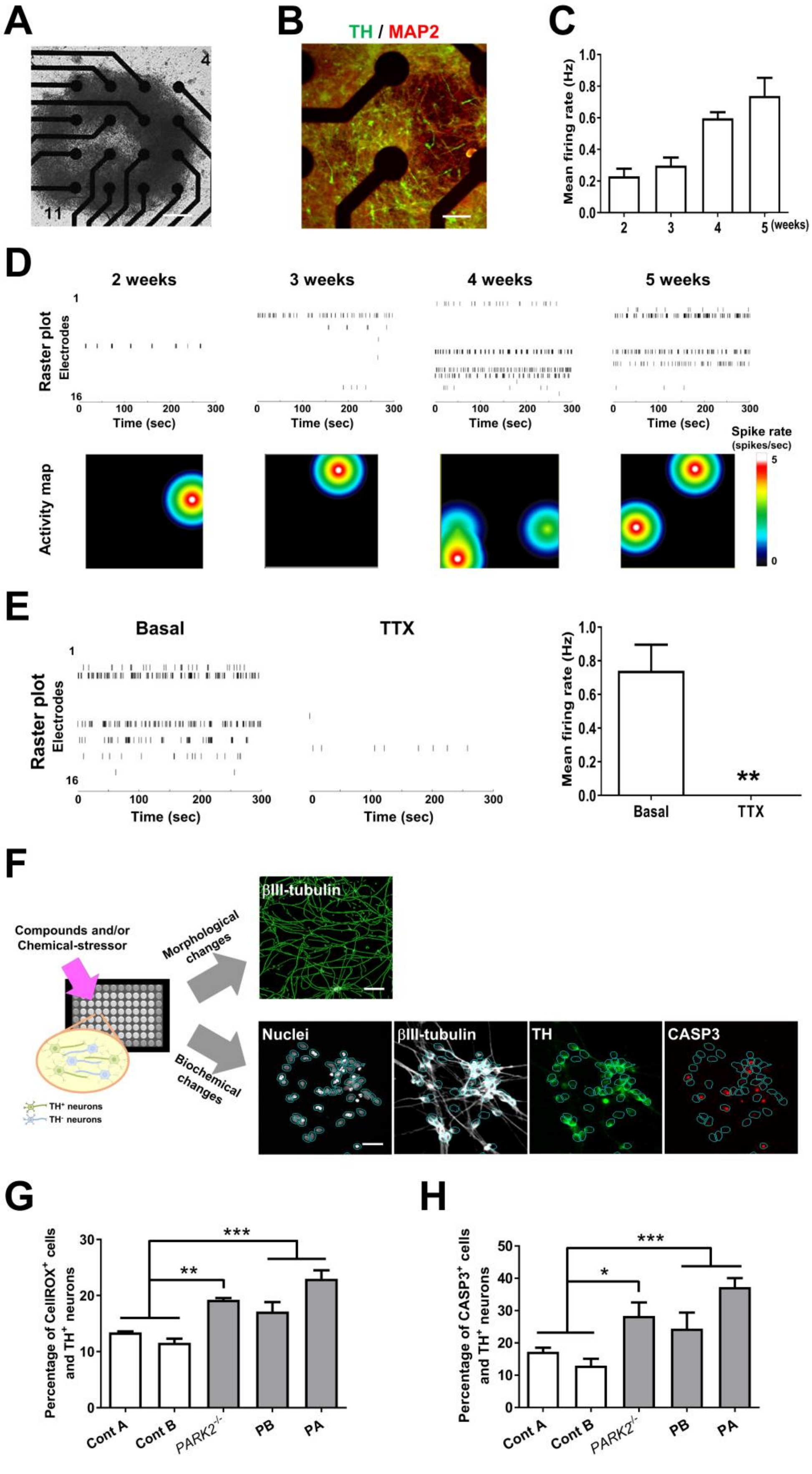
A
4
11
B
TH / MAP2
C
Mean firing rate (Hz)
2
3
4
5 (weeks)
D
2 weeks
3 weeks
4 weeks
5 weeks
Raster plot
Electrodes
Time (sec)
Spike rate (spikes/sec)
Activity map
E
Basal
TTX
Raster plot
Electrodes
Time (sec)
Mean firing rate (Hz)
**
F
Compounds and/or Chemical-stressor
Morphological changes
Biochemical changes
TH+ neurons
TH- neurons
βIII-tubulin
Nuclei
TH
CASP3
G
Percentage of CellROX+ cells and TH+ neurons
Cont A
Cont B
PARK2-/-
PB
PA
H
Percentage of CASP3+ cells and TH+ neurons

Figure S2. Characterization of Cultured iPSC-derived Dopaminergic Neurons Using a MEA System and High-Content Image Analysis (Related to Figure 1)

(A and B) Spontaneous firing of cultured iPSC-derived DA neurons (Control B) using a MEA system was determined. Representative images of DA neurons on Day 34. Bright-field image. Scale bar = 300 μm (A). Immunocytochemical staining. Scale bar = 100 μm (B). (C) Quantification of the spontaneous firing rate per active electrode of DA neurons (Control B) using 48-well plate (16 electrodes per well). Data represent the means ± SEM (n = 4 independent biological replicates). (D) Changes in the spontaneous firing pattern of DA neurons (Control B) from 2 to 5 weeks. Representative images of raster plot and activity map in the basal condition. In a raster plot, each row depicts one electrode and every mark represents one spike. (E) The effects of sodium channel blocker tetrodotoxin (TTX) on the mean firing rate. Representative images of raster plot of the DA neurons (Control B) on Day 34 before and after exposure to TTX (10 nM) are shown (left). Quantification is shown (right). Data represent the means ± SEM (n = 4 independent biological replicates). **$p < 0.01$ by an unpaired t-test. (F) Morphological changes were determined by calculating total neurite length per total neuronal cell nucleus. Neurite length of βIII-tubulin$^+$ cells was quantified using an algorithm of the IN Cell Investigator (GE Healthcare). For visualization and quantification of apoptotic cells, DA neurons were immunostained with DAPI, βIII-tubulin, TH and CASP3. For cell segmentation, nuclear areas were defined using DAPI staining. Double positive staining of βIII-tubulin and TH was defined as DA neurons. To measure the intracellular oxidative stress of DA neurons, the cells were incubated with CellROX Green. The viability of DA neurons (or intracellular oxidative stress levels) was determined by the proportion of CASP3$^+$ (or CellROX Green$^+$) cells among DA neurons. Scale bar = 100 μm (upper) and 20 μm (lower). (G) Elevated intracellular oxidative stress in PARK2- and *PARK2*$^{-/-}$-DA neurons. Intracellular oxidative stress was assessed in DA neurons on Day 14 with an indicator, CellROX. Data represent the means ± SEM (n = 3 independent biological replicates). **$p < 0.01$, ***$p < 0.001$ by Dunnett's multiple comparison test. (H) The abundance of apoptotic cells was increased in PARK2- and *PARK2*$^{-/-}$-DA neurons on Day 14. Data represent the means ± SEM (n = 7-10 independent biological replicates). *$p < 0.05$, ***$p < 0.001$ by Dunnett's multiple comparison test.

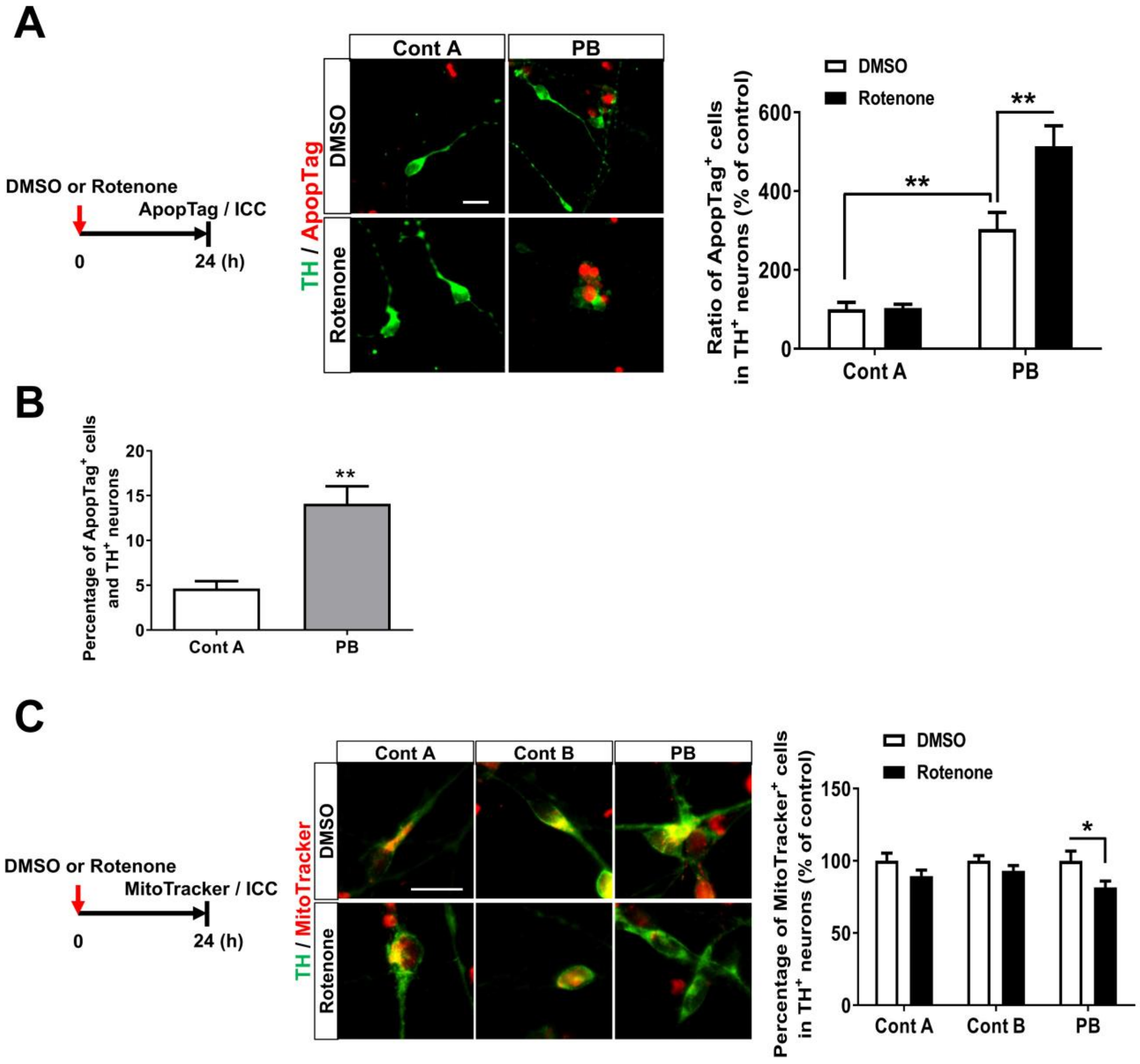
A
DMSO or Rotenone
ApopTag / ICC
0
24 (h)
TH / ApopTag
Cont A
PB
DMSO
Rotenone
DMSO
Rotenone
Ratio of ApopTag+ cells in TH+ neurons (% of control)
600
400
200
0
**
**
Cont A
PB
B
Percentage of ApopTag+ cells and TH+ neurons
20
15
10
5
0
**
Cont A
PB
C
DMSO or Rotenone
MitoTracker / ICC
0
24 (h)
TH / MitoTracker
Cont A
Cont B
PB
DMSO
Rotenone
DMSO
Rotenone
Percentage of MitoTracker+ cells in TH+ neurons (% of control)
150
100
50
0
*
Cont A
Cont B
PB

Figure S3. PARK2 iPSC-derived Dopaminergic Neurons Showed Increased Susceptibility to Rotenone-induced Stress (Related to Figures 1 and 2)

(A) Immunocytochemical analysis of ApopTag^{+} cells in the control (Control A) and PARK2 (PB)-DA neurons on Day 14 with or without rotenone treatment (10 μM, 24 h) using the TUNEL method. Representative staining images of ApopTag^{+} cells are shown (middle). Quantification of apoptotic cells in TH^{+} neurons is shown (right). Data represent the means ± SEM (n = 3-6 independent biological replicates). **$p < 0.01$ by Tukey's multiple comparison test. Scale bar = 20 μm. (B) The abundance of apoptotic cells was increased in PARK2 (PB)-DA neurons on Day 14. Data represent the means ± SEM (n = 6 independent biological replicates). **$p < 0.01$ by an unpaired t-test. (C) Reduced the MitoTracker^{+} fraction by rotenone treatment in PARK2(PB)-DA neurons on Day 14 compared with the control (Control A and B). Representative staining images of MitoTracker^{+} cells with or without rotenone exposure (10 μM, 24 h) are shown (middle). Quantification of MitoTracker^{+} cells is shown (right). Data represent the means ± SEM (n = 3-6 independent biological replicates). *$p < 0.05$ by a t-test with Sidak's correction. Scale bar = 20 μm.

A

Compound	Description	Selectivity of Ca^{2+} channels
Benidipine	a CCA	L, N, T
Cinnarizine	a CCA and antihistamine	L, T
Amiodarone	a CCA, antianginal and antiarrhythmic agent	L, T
Suloctidil	a CCA	Non-selective
Proscillaridin	a cardiac glycoside	-
Tyrothricin	an antibiotic	-
Bacampicillin	an antibiotic	-
Ivermectin	a antiparasite medication	-
Chlorhexidine	a disinfectant and topical anti-infective agent	-

CCA, Ca^{2+} channel antagonist

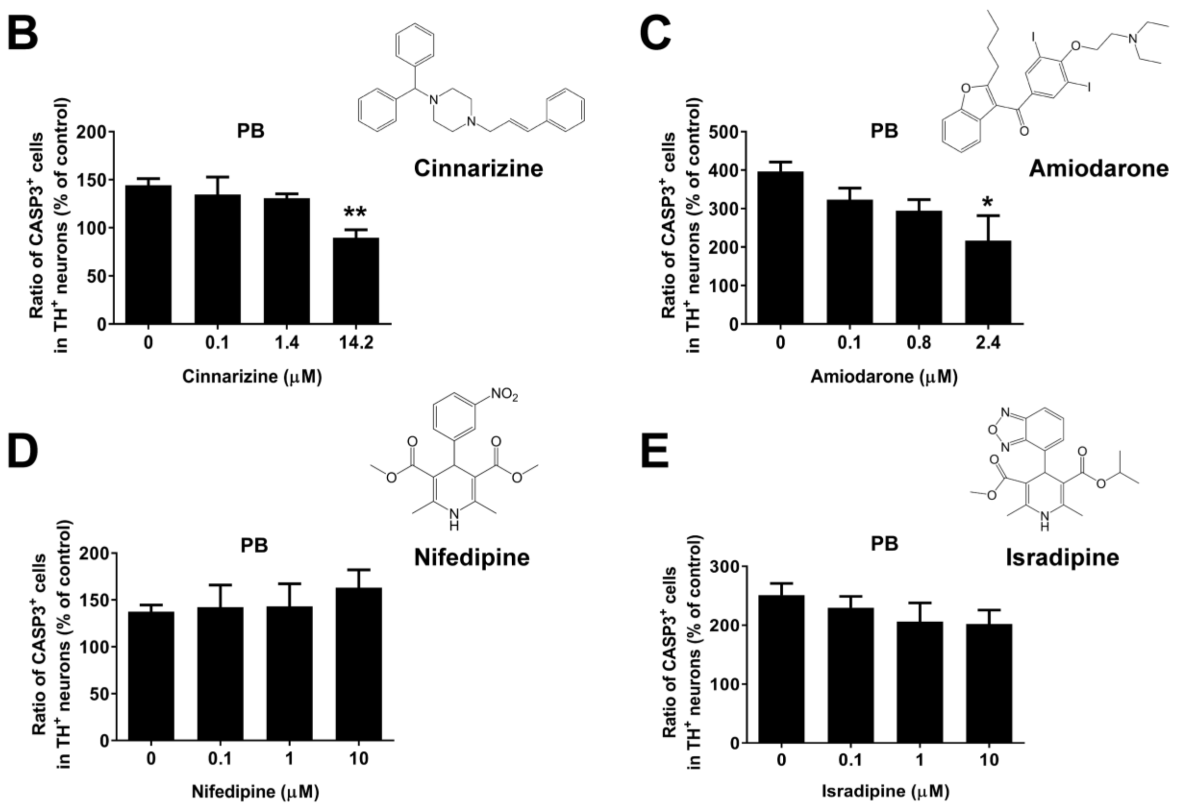

Figure S4. Categories of 9 Compounds Confirmed as Hits by a Concentration Dependency Assay (Related to Figure 3)

(A) The compounds were categorized based on their potential effects. (B-E) Protective effects of cinnarizine, amiodarone, nifedipine and isradipine on rotenone-mediated (10 μM, 24 h) apoptosis. Immunocytochemical analysis of $CASP3^{+}$ cells in PARK2 (PB)-DA neurons was conducted with cinnarizine (B), amiodarone (C), nifedipine (D) and isradipine (E). Data represent the means ± SEM (n = 3-12 independent biological replicates). $*p < 0.05$, $**p < 0.01$ by Dunnett's multiple comparison test.

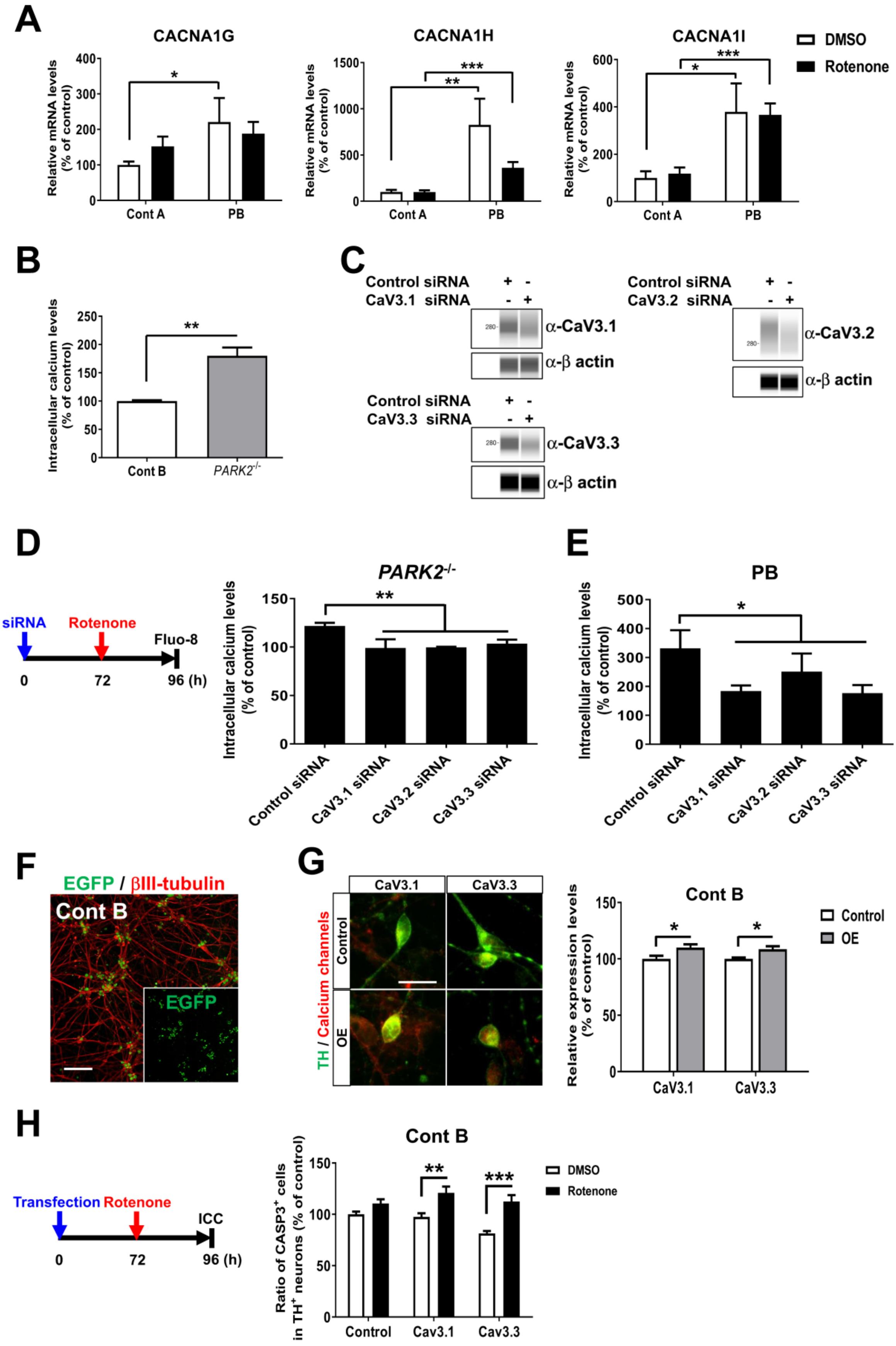
A
CACNA1G
CACNA1H
CACNA1I
DMSO
Rotenone
Relative mRNA levels (% of control)
Cont A
PB
B
Intracellular calcium levels (% of control)
Cont B
PARK2-/-
C
Control siRNA
CaV3.1 siRNA
α-CaV3.1
α-β actin
Control siRNA
CaV3.2 siRNA
α-CaV3.2
α-β actin
Control siRNA
CaV3.3 siRNA
α-CaV3.3
α-β actin
D
siRNA
Rotenone
Fluo-8
0
72
96 (h)
PARK2-/-
Intracellular calcium levels (% of control)
Control siRNA
CaV3.1 siRNA
CaV3.2 siRNA
CaV3.3 siRNA
E
PB
Intracellular calcium levels (% of control)
Control siRNA
CaV3.1 siRNA
CaV3.2 siRNA
CaV3.3 siRNA
F
EGFP / βIII-tubulin
Cont B
EGFP
G
CaV3.1
CaV3.3
Control
OE
TH / Calcium channels
Cont B
Relative expression levels (% of control)
Control
OE
CaV3.1
CaV3.3
H
Transfection
Rotenone
ICC
0
72
96 (h)
Cont B
Ratio of CASP3+ cells in TH+ neurons (% of control)
DMSO
Rotenone
Control
Cav3.1
Cav3.3

Figure S5. Dysregulation of Calcium Homeostasis in Dopaminergic Neurons by T-type Calcium Channels Contributed to Disease-related Phenotypes of PD (Related to Figure 5)

(A) The qRT-PCR reaction was performed on Day 14 for the T-type calcium channel subtypes (CACNA1G (CaV3.1), CACNA1H (CaV3.2) and CACNA1I (CaV3.3)). Data represent the means ± SEM (n = 4-8 independent biological replicates). $*p < 0.05$, $**p < 0.01$, $***p < 0.001$ by an unpaired t-test. (B) Measurement of intracellular calcium levels of neurons (Control B and *PARK2*$^{-/-}$) on Day 14 by the fluorescent calcium indicator Fluo-8 AM. Data represent the means ± SEM (n = 4 independent biological replicates). $**p < 0.01$ by an unpaired t-test.

(C) Expression of T-type calcium channel subtypes after siRNA transfection was examined using subtype specific antibodies. An antibody against β actin is used as an internal control. (D and E) The involvement of T-type calcium channel subtypes in the regulation of intracellular calcium levels was examined in the *PARK2*$^{-/-}$- and PARK2 (PB)-derived DA neurons on Day 14 with rotenone treatment (10 μM, 24 h). The cells were transfected with siRNA against the various T-type calcium channel subtypes for 72 h prior to rotenone treatment. Data represent the means ± SEM (n = 3 independent biological replicates). $*p < 0.05$, $**p < 0.01$ by an unpaired t-test. (F) Representative image of EGFP^{+} cells in βIII-tubulin^{+} neurons after 72 h of transfection with the CMV-EGFP plasmid in DA neurons (Control B) using Lipofectamine 3000. After transfection, immunocytochemical staining was performed with antibody for βIII-tubulin. Insets are the zoomed-out images of EGFP^{+} signals. Scale bar = 100 μm. (G) Immunocytochemistry was performed in DA neurons (Control B) after overexpression (OE) of the T-type calcium channel subtypes (CaV3.1 or CaV3.3). Representative image of calcium channel subtypes in DA neurons (left). The relative expression levels of calcium channel subtypes are shown (right). Data represent the means ± SEM (n = 6 independent biological replicates). $*p < 0.05$ by a t-test with Sidak's correction. (H) Immunocytochemical analysis of CASP3^{+} cells in DA neurons (Control B) transfected for 72 h with the CMV-T-type calcium channel subtypes (CaV3.1 or CaV3.3) plasmid on Day 14 with or without rotenone exposure (10 μM, 24 h). Data represent the means ± SEM (n = 6 independent biological replicates). $**p < 0.01$, $***p < 0.001$ by Tukey's multiple comparison test.

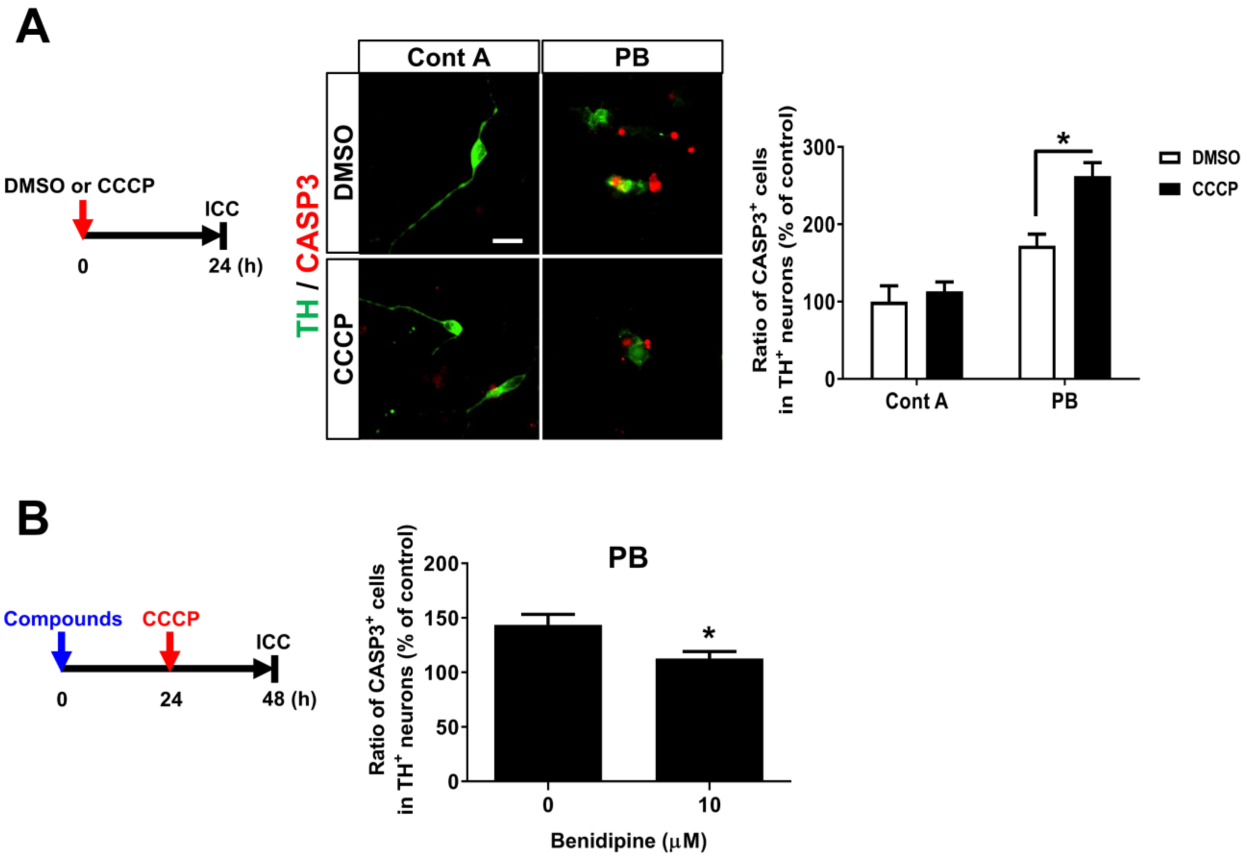

A
DMSO or CCCP
ICC
0
24 (h)
Cont A
PB
DMSO
CCCP
TH / CASP3
Ratio of CASP3+ cells in TH+ neurons (% of control)
300
200
100
0
*
DMSO
CCCP
Cont A
PB
B
Compounds
CCCP
ICC
0
24
48 (h)
PB
Ratio of CASP3+ cells in TH+ neurons (% of control)
200
150
100
50
0
*
0
10
Benidipine (μM)

Figure S6. Calcium Channel Antagonist Protected against Mitochondrial Oxidative Phosphorylation Uncoupler-induced Apoptosis in PARK2 iPSC-derived Dopaminergic Neurons (Related to Figures 2, 3 and 6)

(A) Immunocytochemical analysis of CASP3$^+$ cells in the control (Control A) and PARK2 (PB)-DA neurons on Day 14 with or without carbonyl cyanide m-chlorophenylhydrazone (CCCP) treatment (10 μM, 24 h). Representative images of CASP3 immunostaining are shown (left). Scale bar = 20 μm. Quantification is shown (middle). Data represent the means ± SEM (n = 3 independent biological replicates). *$p < 0.05$ by Tukey's multiple comparison test. (B) Protective effects of benidipine on CCCP-mediated (10 μM, 24 h) apoptosis. Data represent the means ± SEM (n = 6 independent biological replicates). *$p < 0.05$ by an unpaired t-test.

Table S1. Antibody list used in this study

Antibody	Dilution	Host	Catalogue number	Supplier	Location
Primary antibodies;					
anti-SOX1	1:1000	Goat	AF3369	R&D Systems	Minneapolis, MN
anti-SOX2	1:500	Mouse	MAB2018	R&D Systems	Minneapolis, MN
anti-DACH1	1:200	Rabbit	10914-1-AP	Proteintech	Rosemont, IL
anti-nestin	1:500	Mouse	MAB5326	Merck Millipore	Billerica, MA
anti-βIII-tubulin	1:1000	Mouse	T8660	Sigma-Aldrich	St. Louis, MO
anti-MAP2	1:500	Rabbit	AB5622	Merck Millipore	Billerica, MA
anti-TH	1:1000	Sheep	AB1542	Merck Millipore	Billerica, MA
anti-TH	1:500	Rabbit	AB152	Merck Millipore	Billerica, MA
anti-GFAP	1:500	Rat	13-0300	Thermo Fisher Scientific	Waltham, MA
anti-FOXA2	1:200	Mouse	H00003170-M01	Abnova	Taipei, Taiwan
anti-EN1	1:500	Mouse	4G11	Developmental Studies Hybridoma Bank	Iowa, IA
anti-NURR1	1:500	Mouse	PP-N1404-00	R&D Systems	Minneapolis, MN
anti-GIRK2	1:400	Rabbit	APC-006	Alomone Laboratories	Jerusalem, Israel
anti-cleaved caspase-3	1:1000	Rabbit	9661	Cell Signaling Technology	Danvers, MA
anti-CaV3.1	1:200	Rabbit	ACC-021	Alomone Laboratories	Jerusalem, Israel
anti-CaV3.2	1:200	Rabbit	ACC-025	Alomone Laboratories	Jerusalem, Israel
anti-CaV3.3	1:200	Rabbit	ACC-009	Alomone Laboratories	Jerusalem, Israel
Secondary antibodies;					
Alexa Fluor 488-conjugated anti-sheep IgG	1:500	Donkey	A11015	Thermo Fisher Scientific	Waltham, MA
Alexa Fluor 568-conjugated anti-sheep IgG	1:500	Donkey	A21099	Thermo Fisher Scientific	Waltham, MA
Cy2-conjugated anti-rabbit IgG	1:500	Donkey	711-225-152	Jackson Immuno Research	West Grove, PA
Cy2-conjugated anti-mouse IgG	1:500	Donkey	715-225-150	Jackson Immuno Research	West Grove, PA
Cy3-conjugated anti-goat IgG	1:500	Donkey	705-165-147	Jackson Immuno Research	West Grove, PA
Cy3-conjugated anti-mouse IgG	1:500	Donkey	715-165-150	Jackson Immuno Research	West Grove, PA
Cy3-conjugated anti-rat IgG	1:500	Donkey	712-165-153	Jackson Immuno Research	West Grove, PA
Cy3-conjugated anti-rabbit IgG	1:500	Donkey	711-165-152	Jackson Immuno Research	West Grove, PA
Cy5-conjugated anti-mouse IgG	1:500	Donkey	715-175-151	Jackson Immuno Research	West Grove, PA

DACH1, dachshund homolog 1; MAP2, microtuble-associated protein 2; TH, tyrosine hydroxylase; GFAP, glial fibrillary acidic protein; FOXA2, forkhead box protein A2; EN1, engrailed-1; NURR1, nur-related factor 1; GIRK2, G-protein-activated inwqrd rectifier potassium channel 2

Table S2. Compound list used in this study

Compound	Molecular weight	Catalogue number	Supplier	Location
Benidipine	542.02	B6813	Sigma-Aldrich	St. Louis, MO
Cinnarizine	368.51	C5270	Sigma-Aldrich	St. Louis, MO
Amiodarone	681.77	A8423	Sigma-Aldrich	St. Louis, MO
Suloctidil	337.56	S9384	Sigma-Aldrich	St. Louis, MO
Proscillaridin	530.65	P2428	Sigma-Aldrich	St. Louis, MO
Tyrothricin	1228.44	T3000000	Sigma-Aldrich	St. Louis, MO
Bacampicillin	501.98	1047300	U. S. Pharmacopeia	Rockville, MD
Ivermectin	875.09	090-05521	Wako	Osaka, Japan
Chlorhexidine	578.40	220557	Calbiochem	San Diego, CA
Nifedipine	346.33	N7634	Sigma-Aldrich	St. Louis, MO
Isradipine	371.39	I6658	Sigma-Aldrich	St. Louis, MO
ML218	369.33	SML0385	Sigma-Aldrich	St. Louis, MO
Rotenone	394.42	R8875	Sigma-Aldrich	St. Louis, MO
TTX	319.27	4368-28-9	Wako	Osaka, Japan
CCCP	204.62	C2759	Sigma-Aldrich	St. Louis, MO
DMSO	78.13	D5879	Sigma-Aldrich	St. Louis, MO

DMSO, dimethyl sulfoxide; TTX, tetrodotoxin; CCCP, carbonyl cyanide m-chlorophenylhydrazone

Supplemental Experimental Procedures

Generation of Patient-Specific and Isogenic iPSC-derived Neural Progenitor Cells

The iPSC line 201B7 (Takahashi et al., 2007) (a gift from Dr. Shinya Yamanaka), PARK2 iPSC lines PA9 and PB2 (Imaizumi et al., 2012) and PARK6 iPSC line PKB3 (Shiba-Fukushima et al., 2017) were established and maintained as previously described. A PARK2-deficient iPSC line (B7PA21) was generated by applying CRISPR-Cas9 to 201B7 cells (Figure S1) (Suda et al., 2018). In brief, a targeting donor DNA plasmid (pUC- 5'3'PARK2-PurTK) was used to disrupt exon 2 of *PARK2* gene by homologous recombination. The CSIV-U6-*PARK2* (Ex2)-sgRNA-L&R-EF-Csy4-2A-Cas9 was used as a house-made all-in-one vector. The 201B7 was suspended in Opti-MEM (Thermo Fisher Scientific, Waltham, MA) containing Y-27632 (Wako, Tokyo, Japan), house-made all-in-one vector and targeting donor DNA vector plasmid. Electroporation of plasmid DNA was performed using a NEPA21 electroporator (Nepa Gene Co., Ichikawa, Japan). As shown in Figure S1B, homologous recombination in the *PARK2* gene was confirmed by Southern blotting analysis. Furthermore, the expression levels of Parkin was determined by the capillary electrophoresis immunoassay (Figure S1C). The iPSC-derived NPC cell line AF22 (Falk et al., 2012) was kindly provided by Dr. Austin Smith. NPCs were established from the 201B7, PA9, PB2, PKB3, and B7PA21 iPSC lines as previously described (Falk et al., 2012).

Cell Culture

iPSC-derived NPC cultures were maintained in N2 media supplemented with 10 ng/mL epidermal growth factor (EGF; Peprotech, Rocky Hill, NJ) and 10 ng/mL basic fibroblast growth factor (bFGF; StemCultures, Rensselaer, NY) as previously described (Falk et al., 2012). To generate midbrain DA neurons, we cultured the NPCs in N2 media supplemented with 200 ng/mL FGF8 (Peprotech) and 200 ng/mL sonic hedgehog (SHH; R&D Systems, Minneapolis, MN) for 7 days and then further cultured them for 7 days with FGF8, SHH and 1 μM CHIR99021 (CHIR; Stemgent, Cambridge, MA) in Matrigel (BD Biosciences, Franklin Lakes, NJ)-coated dishes. To consistently induce DA neurons, we cryopreserved cells that had been pretreated with FGF8 and SHH for 7 days, using Cell Banker-2 (ZENOAQ, Fukushima, Japan). After midbrain patterning treatment the cells were allowed to differentiate into DA neurons in KBM Neural Stem Cell media (KohjinBio, Saitama, Japan) supplemented with B27 (1:50; Thermo Fisher Scientific)

(referred to as MHM media) along with 20 ng/mL brain-derived neurotrophic factor (BDNF; R&D Systems), 20 ng/mL glial-derived neurotrophic factor (GDNF; Peprotech), 1 ng/mL transforming growth factor-β3 (TGF-β3; R&D Systems), 200 μM ascorbic acid (A.A.; Sigma-Aldrich, St. Louis, MO), 500 μM dibutyryl-cAMP (db-cAMP; Sigma-Aldrich) and 2 μM DAPT (Sigma-Aldrich) (referred to as differentiation media) and seeded at a density of 50,000 cells/cm^2 on 96-well plates coated with poly-D-lysine (PDL; Greiner Bio-One, Kremsmünster, Austria) and 10 μg/mL laminin (Thermo Fisher Scientific). The medium was changed every 3 or 4 days.

Measurement of Dopamine Release

Dopamine release was quantified using a Dopamine Research ELISA Kit (Labor Diagnostika Nord, Nordhorn, Germany) in samples cultured for 3 days at each stage of differentiation, such as 2, 3, and 4 weeks (Figures 1E and 1F). The cells at the last step of differentiation into DA neurons were plated at a density of 200,000 cells/cm^2. The assay was performed following the manufacturer's instructions. Absorbance was read using an iMark microplate reader (BIO-RAD, Hercules, CA) set to 450 nm with a reference wavelength of 595 nm.

Microelectrode Array Recording

A microelectrode array (MEA) recording was performed using the Maestro system (Axion Biosystems, Atlanta, GA) as previously described (Isoda et al., 2016). To prepare iPSC-derived DA neurons for array recording, midbrain patterning treated cells were seeded at a density of 100,000 cells/well on 48-well plates (CytoView MEA) coated with 100 μg/mL PDL (Sigma-Aldrich) and 10 μg/mL laminin (Figures S2A-S2E). A detection threshold was set to +6.0× standard deviation of the baseline electrode noise. Spike raster plots were analyzed using a Neural Metric Tool (Axion Biosystems). The active electrode criterion was defined as an electrode having an average of more than 5 spikes/min. To ascertain the biological nature of the recordings, the sodium channel blocker tetrodotoxin (Wako) was added to the medium, and neuronal activity was recorded (Figure S2E) (Du et al., 2016).

Immunocytochemistry

Immunocytochemistry was performed as previously described (Tabata et al., 2015). The primary antibodies and secondary antibodies used in this study are listed in Table S1. Dopamine was detected using a STAINperfect Immunostaining Kit (rabbit anti-dopamine; 1:500; ImmuSmol, Pessac, France) (Monzel et al., 2017) according to the manufacturer's protocol.

High-Content Analysis

Images were obtained using an IN Cell Analyzer 6000 (GE Healthcare, Chicago, IL). A set of 20 fields per well were scanned at 20× magnification, and image analysis was conducted with the IN Cell Developer Toolbox 1.9 software (GE Healthcare) using custom-developed analysis protocols. For cell segmentation and the nucleus counts, images were acquired using DAPI staining (Figures S2F-S2H). In the neural cell population analysis, we divided the number of cells positive for each marker by the total number of cells (stained with DAPI). The neurite arborization of βIII-tubulin^{+} neurons was quantified using an algorithm in the IN Cell Developer Toolbox 1.9. Morphological changes were determined by calculating the total neurite length per total neuronal cell. For the visualization and quantification of intracellular oxidative stress or mitochondria, the cells were incubated with CellROX Green (1 μM, Thermo Fisher Scientific) or MitoTracker Red CMXRos (0.5 μM, Thermo Fisher Scientific) for 30 min at 37°C and 5% CO_2 incubated with MHM media, respectively. Thereafter, immunostaining for βIII-tubulin and TH was performed. The intracellular oxidative stress levels or mitochondria were determined by the proportion of CellROX^{+} cells or MitoTracker^{+} cells (Figure S3C) among DA neurons. To measure the viability of DA neurons, we quantified apoptotic cells by immunohistochemistry for CASP3. In addition, we used an ApopTag Fluorescein In Situ Apoptosis Detection Kit (sheep anti-digoxigenin fluorescein; Merck Millipore, Billerica, MA) based on the terminal deoxynucleotidyl transferase dUTP nick end labeling (TUNEL) method according to the manufacturer's protocol as an alternative method to examine apoptosis (Figures S3A and S3B).

Compound Screening

False positive compounds among the primary hit compounds were excluded by concentration-dependency assays. The results of the primary screening were made into a dot plot by the software TIBCO Spotfire (TIBCO Software, Palo Alto, CA).

Calcium Imaging

The cells were incubated with a fluorescent calcium indicator Fluo-8 acetoxymethyl (AM; 5 µM, AAT Bioquest, Sunnyvale, CA) in Hank's Balanced Salt Solution (Nacalai Tesque) with Pluronic F-127 (0.02%; Dojindo, Kumamoto, Japan) and Hoechst-33342 (2 µg /mL; Sigma-Aldrich) for 30 min at 37°C and 5% CO_2. To assess the baseline calcium levels, we immediately scanned the cells using an IN Cell Analyzer 6000. A set of 4 fields per well were scanned at 10× magnification. Subsequently, the cells were incubated with a calcium ionophore, A23187 (4 µM; Tocris Bioscience, Bristol, UK), for 30 min and further incubated with a calcium-selective chelating agent, EGTA (10 mM; Dojindo), for 30 min. The intracellular calcium concentration was calculated using the following equation:

Intracellular calcium level = K_D [(R - R_{min}) / (R_{max} - R)]

The dissociation constant (K_D) value was 389 nM, as described in the manufacturer's protocol. The R_{min} value was obtained in the presence of both A23187 and EGTA. The R_{max} was obtained in the presence of A23187 only. For cell segmentation and nucleus counts, images were acquired using Hoechst-33342 staining.

Gene Silencing with siRNA

DA neurons were incubated for 72 h with Accell siRNA (1 µM, Thermo Fisher Scientific) in a 50:50 mix of Accell delivery media and differentiation media. The Accell siRNAs were Accell Non-targeting siRNA #1 (#D-001910-10), CaV3.1 (#E-006127-00), CaV3.2 (#E-006128-00), and CaV3.3 (#E-006129-00) (Figure 5D). A quantitative reverse transcription polymerase chain reaction (qRT-PCR) was performed to measure the level of gene expression 72 h after the transfection using the Cells to CT Kit (Thermo Fisher Scientific). The qRT-PCR reaction was performed in duplicate using TaqMan gene expression assays (Thermo Fisher Scientific) for GAPDH (Hs02758991_g1), CaV3.1 (Hs00367969_m1), CaV3.2 (Hs01103527_m1), and CaV3.3 (Hs01096207_m1) on a ViiA 7 real-time PCR system (Thermo Fisher Scientific) (Figure S5A). Furthermore, the expression level of the T-type calcium channel subtypes was determined by a capillary electrophoresis immunoassay (Figure S5C).

Plasmid DNA Transfection

The plasmid pEGFP-N1 was purchased from Clontech Laboratories (now Takara Bio USA, Mountain View, CA). The hα1Ga (CACNA1G)–pDsRed and a1Ic (CACNA1I)-HE3-pcDNA3 expression plasmid DNA were obtained from Addgene (#45811 and #45810, respectively; Cambridge, MA). Transfection of the plasmid into iPSC-derived DA neurons was performed using Lipofectamine 3000 transfection reagent (Thermo Fisher Scientific) according to the manufacturer's protocol. Immunocytochemistry was performed to measure the level of expression 72 h after the transfection (Figures S5F-S5H).

Chemicals

All chemicals used in this study are listed in Table S2. The chemical structures were drawn using ChemDraw Prime 16.0 (PerkinElmer, Waltham, MA).

Supplemental References

Du, F., Yu, Q., Shijun, Y., Doris, C., and Yan, S.S. (2016). Development and Dynamic Regulation of Mitochondrial Network in Human Midbrain Dopaminergic Neurons Differentiated from iPSCs. Stem Cell Reports. *7*, 678-692.

Falk, A., Koch, P., Kesavan, J., Takashima, Y., Ladewig, J., Alexander, M., Wiskow, O., Tailor, J., Trotter, M., Pollard, S., et al. (2012). Capture of neuroepithelial-like stem cells from pluripotent stem cells provides a versatile system for in vitro production of human neurons. PLoS One. *7*, e29597.

Imaizumi, Y., Okada, Y., Akamatsu, W., Koike, M., Kuzumaki, N., Hayakawa, H., Nihira, T., Kobayashi, T., Ohyama, M., Sato, S., et al. (2012). Mitochondrial dysfunction associated with increased oxidative stress and α-synuclein accumulation in PARK2 iPSC-derived neurons and postmortem brain tissue. Molecular Brain. *5*, 35.

Monzel, A.S., Smits, L.M., Hemmer, K., Hachi, S., Moreno, E.L., van Wuellen, T., Jarazo, J., Walter, J., Brüggemann, I., Boussaad, I., et al. (2017). Derivation of human midbrain-specific organoids from neuroepithelial stem cells. Stem Cell Reports. *8*, 1144-1154.

Shiba-Fukushima, K., Ishikawa, K.I., Inoshita, T., Izawa, N., Takanashi, M., Sato, S., Onodera, O., Akamatsu, W., Okano, H., Imai, Y., et al. (2017). Evidence that phosphorylated ubiquitin signaling is involved in the etiology of Parkinson's disease. Hum Mol Genet.

Suda, Y., Kuzumaki, N., Sone, T., Narita, M., Tanaka, K., Hamada, Y., Iwasawa, C., Shibasaki, M., Maekawa, A., Matsuo, M., et al. (2018). Down-regulation of ghrelin receptors on dopaminergic neurons in the substantia nigra contributes to Parkinson's disease-like motor dysfunction. Mol Brain. *11*, 6.

Tabata, Y., Murai, N., Sasaki, T., Taniguchi, S., Suzuki, S., Yamazaki, K., and Ito, M. (2015). Multiparametric phenotypic screening system for profiling bioactive compounds using human fetal hippocampal neural stem/progenitor cells. Journal of Biomolecular Screening. *20*, 1074-1083.

Takahashi, K., Tanabe, K., Ohnuki, M., Narita, M., Ichisaka, T., Tomoda, K., and Yamanaka, S. (2007). Induction of pluripotent stem cells from adult human fibroblasts by defined factor. Cell. *131*, 861-872.
